# Toolbox for the structure-guided evolution of ferulic acid decarboxylase (FDC)

**DOI:** 10.1038/s41598-022-07110-w

**Published:** 2022-03-01

**Authors:** Horia Duță, Alina Filip, Levente Csaba Nagy, Emma Zsófia Aletta Nagy, Róbert Tőtős, László Csaba Bencze

**Affiliations:** Enzymology and Applied Biocatalysis Research Center, Faculty of Chemistry and Chemical Engineering, Babeș-Bolyai University, Arany János Street 11, 400028 Cluj-Napoca, Romania

**Keywords:** Biocatalysis, Enzymes, Biochemistry, Chemical biology

## Abstract

The interest towards ferulic acid decarboxylase (FDC), piqued by the enzyme’s unique 1,3-dipolar cycloaddition mechanism and its atypic prFMN cofactor, provided several applications of the FDC mediated decarboxylations, such as the synthesis of styrenes, or its diverse derivatives, including 1,3-butadiene and the enzymatic activation of C-H bonds through the reverse carboligation reactions. While rational design-based protein engineering was successfully employed for tailoring FDC towards diverse substrates of interest, the lack of high-throughput FDC-activity assay hinders its directed evolution-based protein engineering. Herein we report a toolbox, useful for the directed evolution based and/or structure-guided protein engineering of FDC, which was validated representatively on the well described FDC, originary from *Saccharomyces cerevisiae* (*Sc*FDC). Accordingly, the developed fluorescent plate-assay allows in premiere the FDC-activity screens of a mutant library in a high-throughput manner. Moreover, using the plate-assay for the activity screens of a rationally designed 23-membered *Sc*FDC variant library against a substrate panel comprising of 16, diversely substituted cinnamic acids, revealed several variants of improved activity. The superior catalytic properties of the hits revealed by the plate-assay, were also supported by the conversion values from their analytical scale biotransformations. The computational results further endorsed the experimental findings, showing inactive binding poses of several non-transformed substrate analogues within the active site of the *wild-type Sc*FDC, but favorable ones within the catalytic site of the variants of improved activity. The results highlight several ‘hot-spot’ residues involved in substrate specificity modulation of FDC, such as I189, I330, F397, I398 or Q192, of which mutations to sterically less demanding residues increased the volume of the active site, thus facilitated proper binding and increased conversions of diverse non-natural substrates. Upon revealing which mutations improve the FDC activity towards specific substrate analogues, we also provide key for the rational substrate-tailoring of FDC.

## Introduction

Ferulic acid decarboxylase (FDC), as member of the carboxy-lyase family (EC 4.1.1.) has attracted significant interest in recent years, mainly due to its unusual, prenylated flavin mononucleotide (prFMN) cofactor^1^, as well as to its unique mechanism, representing the first enzymatic 1,3-dipolar cycloaddition^2,3^.

Besides the studies focusing on the elucidation of the structure and mechanism of FDC, as first application of FDC, the biosynthesis of styrene, involving the decarboxylation of cinnamic acid within engineered *Escherichia coli* cells, has been reported^4,5^. Subsequently, FDC was shown to possess broad substrate tolerance, decarboxylating differently (*orto-*, *meta-* or *para-)* substituted cinnamic acids, as well as its biaryl or heteroaryl derivatives^2,6,7^. Recently, FDC has been employed in an enzymatic cascade yielding stilbenes^8^ or shown to activate C-H bonds through CO_2_ fixation, yielding unsaturated aromatic carboxylic acids^9^. Other studies investigated the effect of mutations/deletions of *fdc1* on the production of volatile phenols obtained during yeast alcoholic fermentation^10^, or quantitatively monitored the fermentation byproducts when *fdc1* in *S. cerevisiae* and *S. eubazanus* have been CRISPR edited^11^. The effect of *fdc1* single nucleotide polymorphisms (SNPs) on the decarboxylation activity of some industrially relevant yeasts was also reported^12^.

The stability issues of the prFMN cofactor^13^, as one the major factors limiting the applicability of FDC reactions, can be alleviated by the use of whole-cell^6,7,14^ or cell-free extract^15^ FDC-biocatalysts. Recently, several studies focused on the biosynthesis of the cofactor, aiming it’s in vivo or in vitro synthesis^16^, that provides access to the fully active, holo-FDC. In *Saccharomyces cerevisiae*, the final step in the cofactor’s biosynthesis, the prenylation of FMN, is catalyzed by PAD1, a flavoprotein that is necessary for the generation of active, holo-FDC. In *E. coli*, UbiX substitutes PAD1 in its role of prFMN’s biosynthesis, that serves as cofactor for UbiD, homologue of FDC^17^. As such, needing both *fdc1* and *pad1* genes for the desired decarboxylase activity^18^, initially FDC has been studied in *S. cerevisiae* cultures, while later it has been shown that *E. coli* cultures expressing only the *fdc1* gene, due to the presence of UbiX of the host *E. coli* cells, can also function as whole-cell biocatalysts with the desired FDC-activity^7,17–20^. Supposedly, the cofactor’s active iminium form is obtained inside FDC’s active site under the influence of oxygen^21^ and a number of conserved residues^22^. However, in solution, prFMNH_2_ can be irreversibly converted to inactive forms, such as prFMN C_4a_-OOH^21^, prFMN-OH, prFMN_radical_, prFMN_radical_-H, prFMN_iminium_-OH, C_1_-ene-prFMN_iminium_^1,13,23,24^, while light decomposure has been also reported^22^, all these hindering the isolation of the holo-FDC.

Despite the cofactor stability issues, the identification of several novel decarboxylases harboring the prFMN cofactor^25–29^, the broad substrate scope of FDC in comparison to phenolic acid decarboxylases from *Enterobacter sp*. or *Bacillus pumilus*^30–32^, or to benzoic acid decarboxylases^33^, that are limited to decarboxylations of 4-OH cinnamates, or of benzoic acids, respectively, propelled FDC as one of the most versatile non-oxidative decarboxylases. Moreover, the high-quality crystal structures of FDC in apo-, holo- and ligand bound variants^1,34–36^ and the elucidation of the 1,3-cycloaddition reaction mechanism^1–3^, allowed initial rational design of FDC towards different substrates of interest, such as aromatic carboxylic acids, bulky cinnamic acid analogues and/or aliphatic substrates^7,9,37,38^.

Encouraged by the versatility of FDC and its increasing biotechnological applicability, herein we provide a toolbox for the efficient laboratory evolution of FDC. Accordingly, we describe the development of a facile, fluorescent cell-plate FDC-activity assay, suitable for an initial, qualitative activity screen of mutant libraries generated through directed evolution or rational design based processes. In this work, the assay was validated by its employment within the activity screens of a focused FDC mutant library towards an aromatic substrate panel of high structural diversity. The increased activities of the FDC variants selected from the plate-assay, were confirmed by analytical scale biotransformations, while computational results supported and revealed the molecular level details of the improved enzyme activities. Notable, that despite the results represent an initial snapshot of employing the developed laboratory evolution toolbox for FDC, the correlation found between the nature/position of a certain functional group of the substrate and the corresponding active-site mutations, that improved the decarboxylation activity, also paves the way for the substrate-tailored protein engineering of FDC.

## Results and discussion

### Generation of substrate panel and focused FDC mutant library

**Figure 1:**
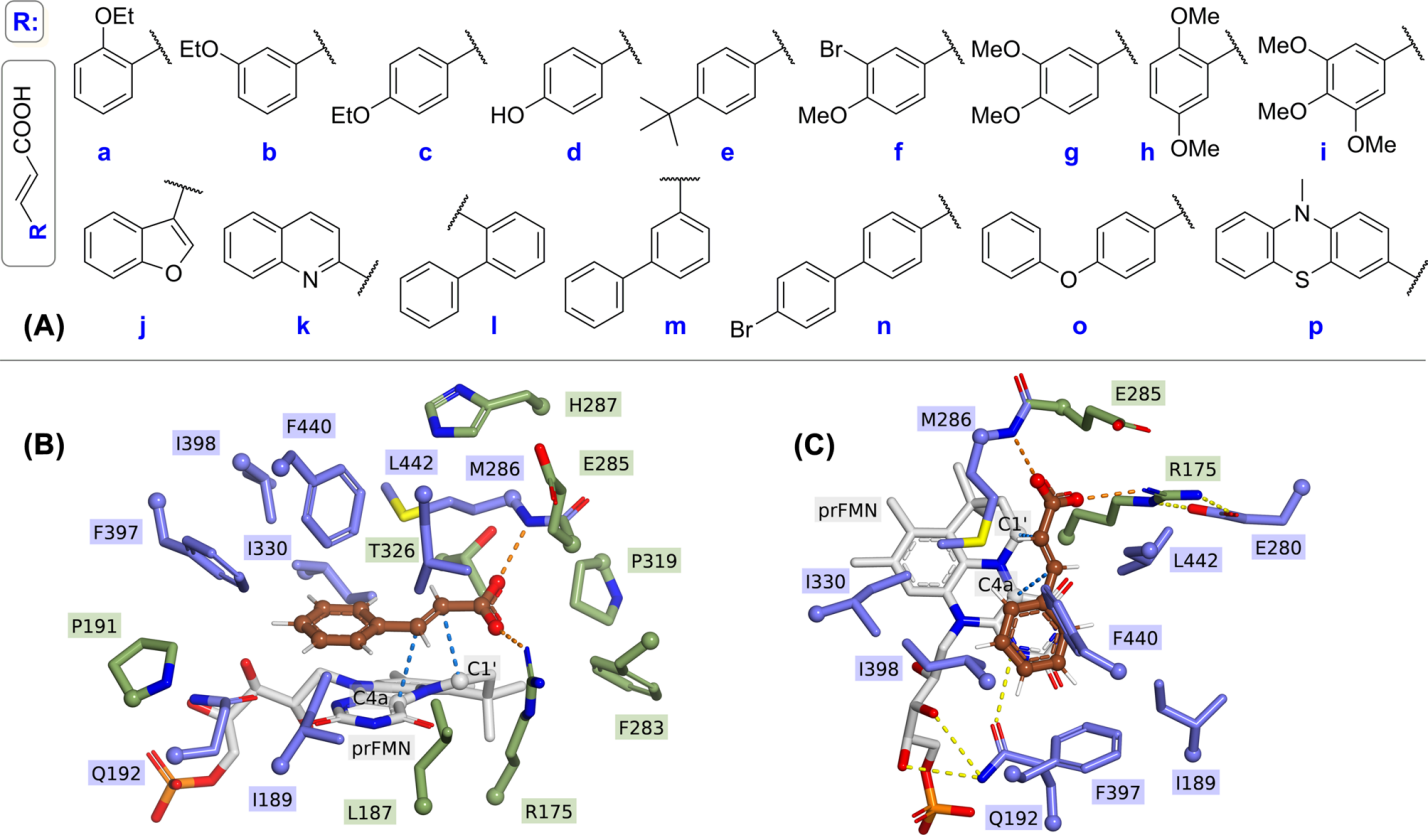
(**A**) substrate panel, including cinnamic acid derivatives **1a**–**p**; (**B**) active site model of *Sc*FDC (PDB: 4ZAC) accommodating *trans*-cinnamic acid as substrate, highlighting in blue the residues selected for individual replacement with alanine/valine (**C**) top view of active site model with respect to the substrate plane: proper substrate binding requires the location of the α − β double bond of the substrate (highlighted in brown) in the proximity of carbon C1’ and C4a atoms of the prFMN cofactor, that facilitates the 1,3-cycloaddition step of the reaction mechanism^1,2^. Hydrogen bonds between residues E280 and R175, as well as Q192 and the cofactor are shown as yellow dashed lines. *Softwares used for the preparation of images are listed in Supporting information, Section 1*.

Among the substrate panel (Fig. 1A) we included substrate analogues with ring-substituents of diverse electronic properties (**1a**–**1e**), or multiple substituted in various, *o*-, *m*-, *p*- positions of the aromatic ring (**1f**–**1i**), as well as heteroaromatic (**1j**, **1k**), or differently connected bulky, biaryl (**1l**–**1o**) or heteroaryl (**1p**) substrate analogues, of which active site orientations in several cases showed steric repulsions with active site residues of *Sc*FDC. Notable, that compound **1p** of the substrate panel was not transformed by *wild-type* FDC^7^, while for bulky biaryl compounds (**1l**–**1o**) no proper active site orientations were obtained by the initial docking predictions. Besides the aim to assess the impact of substrate substitution pattern on the activity of the *wild-type*/mutant FDCs, upon substrate library generation the existing applications of the resulting styrenes were also considered. Accordingly, mono-substituted styrenes **2a**–**2e** are building blocks in the synthesis of biologically active compounds^39,40^, while biaryl or heteroaryl styrenes **2k**, **2l** are relevant for the synthesis of macromolecules^41^ or policyclic aromatic hydrocarbons^42^. Generally, the terminal double bond enables styrenes to act as versatile synthons in a variety of organic synthetic procedures, as reported in case of **2d**–**2i**, **2o**^43–45^. Therefore, the employment/validation of the fluorescence-plate assay within the evolution of FDC towards these substrates intertwines with the synthetic value of the produced styrenes.

Further, we generated a focused *Sc*FDC mutant library (Table S1), by the individual replacement of active site residues to smaller alanine or valine residues (Fig. 1B) to alleviate the steric repulsion of the targeted substrate panel, predicted by the docking studies using our computational model^7^. Residues involved in the reaction mechanism or cofactor/ substrate fixation, such as R175, E280 and E285 were not selected for mutations. Residue E285 is directly involved in the decarboxylation mechanism, having a role in the protonation step of the adduct obtained from the 1,3-dipolar cycloaddition between the C1’ and C4a atoms of the prFMN cofactor and the α-β double bond of the substrate (Fig. 1C), step supposed to precede the Grob-type decarboxylative fragmentation that releases CO_2_^1,2^. Moreover, E285 and R175 interact with the carboxyl group of the substrate, while E280 has a direct influence on the pKa of both R175 and E285 (Fig. 1C). Thus, the active site residues selected for mutations include Q192 and I330, previously found to narrow the active site of *Sc*FDC, hindering the accommodation of bulky non-linear substrates^7^. Since Q192 is also involved in cofactor binding through hydrogen bonding with the ribitol tail of prFMN (Fig. 1C and Fig. S33), besides mutation Q192A, variant Q192N was also considered. The remaining active site residues selected for mutations, such as I189, F397, I398, F440, M286 and L442 outline the hydrophobic region of the active site, accommodating the substrate’s aryl moiety, and while not play an active part in the reaction mechanism, can impose steric constraints on the proper orientation^1,7,22^ of the differently substituted substrates, thus influencing reaction progress.

During the experimental work of our study, other studies implying protein engineering of *An*FDC and *Sc*FDC has been reported, involving several active site residues of *Sc*FDC such as F397, I398, M286 and I330, also selected by us for the mutant library generation. Accordingly, mutation of residues Y394, T395 of *An*FDC and the corresponding homologous residues F397 and I398 of *Sc*FDC provided FDC variants with decarboxylase activity for 1,3-cyclobutadiene^37^. Site-saturation mutagenesis at residues M283 and I327 of *An*FDC (corresponding to M286 and I330 in *Sc*FDC) provided FDC mutants with activity within the decarboxylation of atypic, benzoic acid-type aromatic substrates^9^. Besides these residues, our molecular docking predicted I189 as narrowing the catalytic site, providing steric repulsion in case of substrates with multiple substituted aromatic moieties, while replacement of bulky F440 residue in combination with L442 into smaller hydrophobic residues, was also considered. Notable, that all selected *Sc*FDC active site residues, except F397 and I398 (corresponding to Y394 and T395 of *An*FDC) are conserved within the two highly studied *Sc*FDC and *An*FDC variants (Table S2).

### Cell-plate assay development

While the protein engineering of FDC is of high interest, for an efficient directed evolution-based engineering process, high-throughput activity assays, allowing facile activity screens of largely sized mutant libraries, are highly desirable. A plate assay, suitable for FDC activity screens at whole-cell level, also alleviates the tedious isolation process of holo-FDC^1,19^, however according to our knowledge, no such activity assay has been reported for FDC.

**Figure 2:**
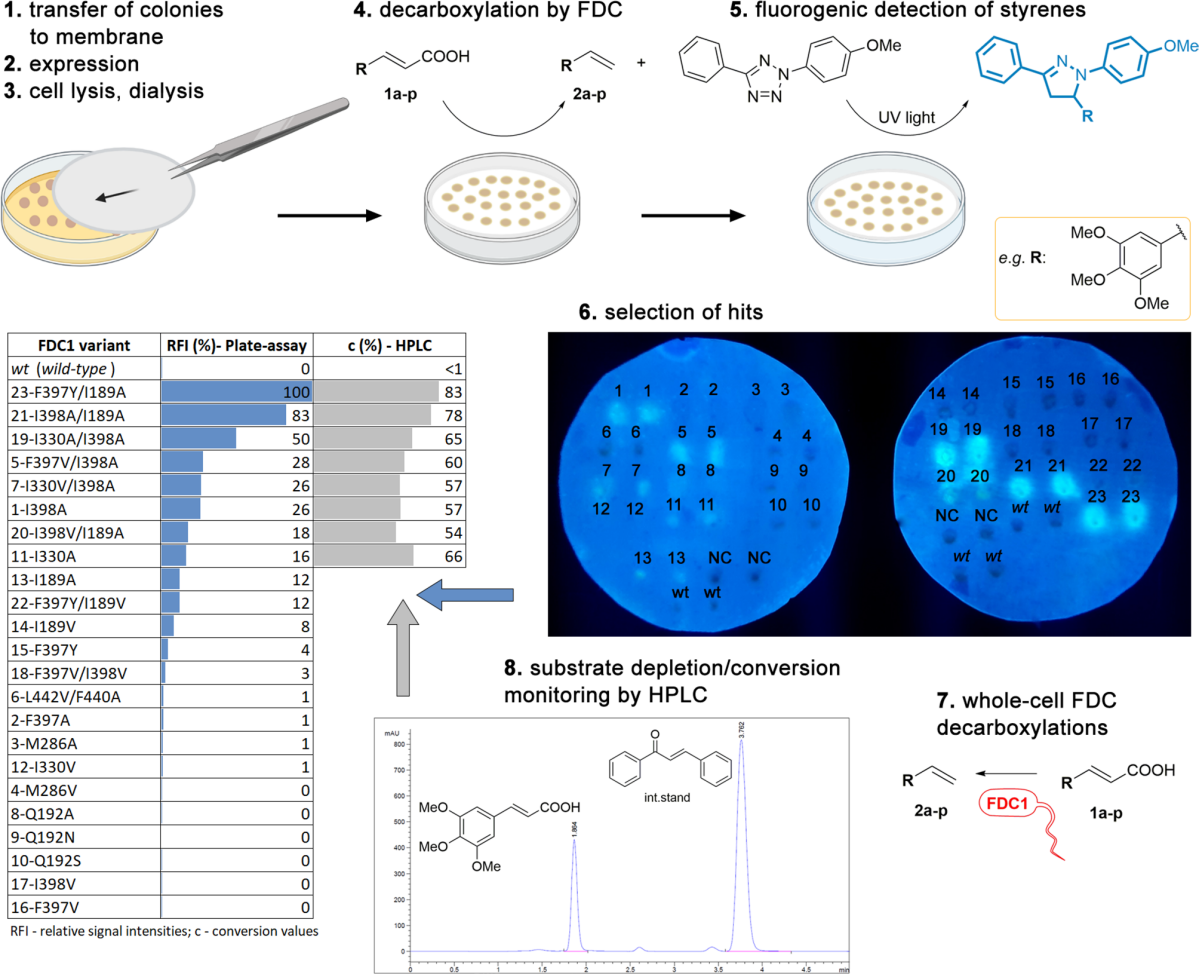
Initial FDC activity screens using the fluorescent cell-plate assay allowing rapid identification of enzyme variants with improved activity, that upon selection have been characterized by the conversion values from their analytical scale decarboxylations. Representative results are presented in case of substrate analogue 3,4,5-trimethoxy-cinnamic acid **1i** (for details of image preparations see *Supplementary Information*, Chapter 6) The cell-plate activity screens has been performed on the whole substrate panel **1a**–**1p** (see Fig. S2–S17 and Tables S3–S5 for results of cell plate assays, while their detailed discussions in Sect. 2.3), while the conversions of the biotransformations of **1a**–**1p** were determined by HPLC (Tables S7–S21) (*Softwares used for image preparation are listed in Supporting information, Section 1)*.

The 1,3-dipolar cycloaddition reaction between an alkene and a tetrazole represents an attractive method of fluorophore-forming bioorthogonal chemistry, with various diaryltetrazoles shown to be highly sensitive fluoroprobes for the detection of alkenes^46–49^. Furthermore, the photoinduced 1,3-dipolar cycloaddition using the selected diaryltetrazole (Fig. 2) as nitrile imine dipole was previously shown to possess compatibility for in vivo protein labeling within *E. coli* whole cells^47–50^. Building on this knowledge, recently, we developed a fluorescent phenylalanine ammonia-lyase (PAL) activity assay, that employs FDC as secondary, reporter enzyme, the produced styrene being fluorescently detected upon its reaction with a tetrazole-based fluorogenic probe^51^. Interestingly, at the same time a similar tetrazole-based assay was also reported for two operation modes of OleT decarboxylase, also validated on a focused mutagenesis library^52^. While both assays were optimized for use of cell-free extracts^51^, herein we focused on adapting the fluorescent detection of styrene derivatives to a cell-plate FDC-activity assay, providing facile, solid-phase decarboxylase activity screens (Fig. 2, steps 1–4).

Within the plate assay whole cells harboring the *fdc1* gene variants of the mutant library were used and tested in the decarboxylation reactions of the entire substrate panel. The plates were performed in duplicates using in each case as negative control, *E. coli* host cells without the plasmid containing *fdc1* gene. In the first step of the assay the cells of the different variants were grown, transferred onto a PVDF membrane, followed by IPTG induced-gene expression (Fig. 2). Cell permeabilization with chloroform, and a subsequent dialysis step of the membrane-attached colonies was followed by the decarboxylation reactions, performed by placing the membrane onto the plate containing 2 mM of the corresponding substrates **1a**–**1p**, followed by incubation at 37 °C for 4 h. For the subsequent fluorogenic reaction, incubation of the membrane with the tetrazole fluoroprobe and UV-irradiation was employed, followed by the detection of the fluorescence signal through an imaging system (Fig. 2).

### FDC-activity screens and HPLC conversions

**Figure 3:**
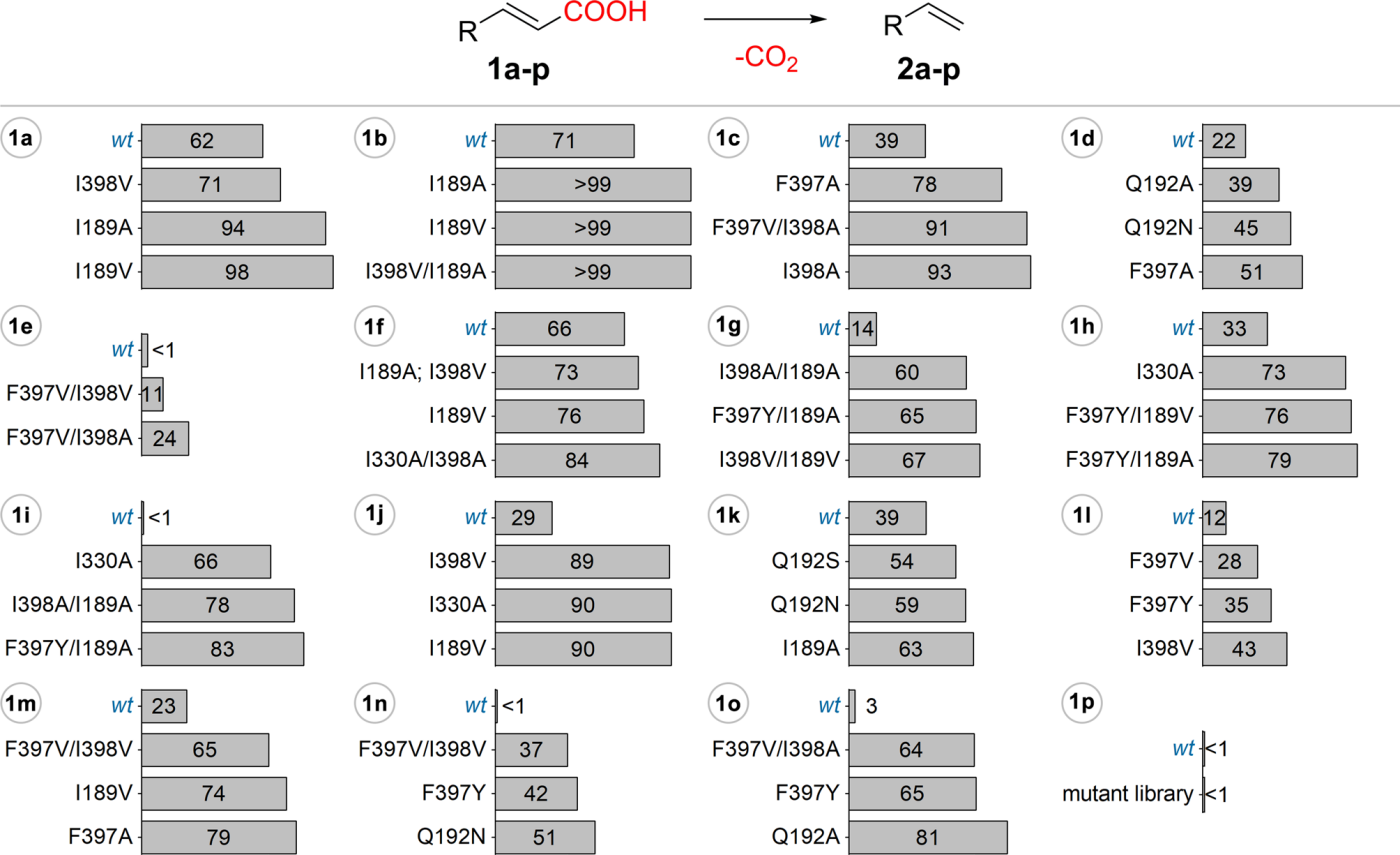
HPLC conversion values obtained from the analytical scale biotransformation of the substrate panel **1a**–**1p**, using *Sc*FDC whole-cell biocatalyst. Results of the best performing three variants and of the *wild-type Sc*FDC are shown. *Softwares used for the preparation of images are listed in Supporting information, Section 1*.

The developed cell-plate assay has been employed for the initial, qualitative assessment of the enzyme activities of the FDC mutant library towards the substrate panel, allowing the selection/identification of the best performing variants, based on their relative fluorescence signal intensities (Tables S3–S5, Fig. S2–S17). Further, the selected best performing variants and several non-reporter variants were used as induced whole cell biocatalysts in analytical scale biotransformations of the corresponding substrates, monitoring the conversion values by reverse phase HPLC to provide their conversion-based activity order (Tables S7–S21, Fig. 3). In case of substrates, **1a**–**1f**, **1h**, **1i**, **1l**, **1n** the activity-order of the variants based on the fluorescent signal intensities from the cell-plate assay were in good correlation with the conversion-based activity ranking of the hits selected from mutant library (Fig. 3 and Tables S7–S12, S14, S15, S18, respectively). Accordingly, the best three-four responders from the plate assay provided the highest conversions of substrates **1a**–**1f**, **1h**, **1i**, **1l**, **1n**, while the variants exhibiting no fluorescence signal were also inactive or resulted in low conversions (< 8%) within the HPLC-monitored biotransformations (Tables S7–S12, S14, S15, S18). Among these results, notable, the presence of some exceptions such as the case of variant F397Y/I189A, providing high fluorescent signal intensity (95% RFI) in the plate-assay and moderate conversions (44%) in case of substrate **1f** (Table S12), or variant I330A with low fluorescent intensity (16% RFI) and high conversion values of 66% in case of **1i** (Table S15). Besides, moderate correlation has been observed in case of substrates **1k** (Table S17), the good reporter variants (RFI > 50%) providing the highest conversions values of > 52%), but moderate reporter variants M3 and M1 (18% and 21% RFI) showed lower conversion values (< 6%) than the weak reporter variant M17 (12% conversion and RFI of 5%). While in case of substrates **1g**, **1j**, **1m**, and **1o** no clear correlation of the activity-order could be observed, the cell-plate assay still provided a suitable “yes/no”-type response for initial activity screens using substrates **1g**, **1j** and **1o**, 24 out of 27 colonies, acting as good reporters (RFI > 50%) in the plate assay, showed significantly increased conversions in the analytical scale biotransformations over those obtained by the low/non-reporter colonies (RFI < 10%), (Tables S13, S16, S21). In case of **1m**, variants with improved conversions (M2, M13, M14, M18) could also be identified among the good reporters (RFI > 50%), but M16 acting as non-reporter within the plate assay provided conversion of 17%, while moderate/good reporter variants M9, M19 and M5 (RFI% of 26, 59 and 37, respectively) showed low HPLC conversions of 4–9% (Table S19). The occurrence of similar false positive hits is also observed at the structurally similar, biphenyl derivative **1n**, in which case variants *wt*, M19 and M23 of low to moderate fluorescence intensities of 11–21% RFI, showed no conversions within the biotransformations (Table S20). In these cases, the increased background fluorescence and low substrate solubilities, combined with cell-growth differences of the colonies from the assay-plate might induce the appearance of false positive hits. Finally, in the particular case of **1p**, the high background fluorescence of the phenothiazine moiety, hindered the cell-plate activity screens (Fig. S17), thus the analytical scale biotransformations of **1p** have been performed with the whole mutant library, however none of the *Sc*FDC variants provided detectable conversions.

These results obtained on a diverse set of cinnamic acids support that the plate-assay is suitable for initial, qualitative-type FDC-activity assessments at whole-cell level, required within the high-throughput screens of the directed evolution-based enzyme engineering. While for several substrates **1a**–**1f**, **1h**, **1i**, **1l**, **1n** the activity rank of the mutant library obtained with the plate assay resembled the conversion-based activity order, the low-degree correlation obtained in case of substrates **1g**, **1j**, **1m**, **1o**, recommends the use of the plate assay for activity screens with a “yes/no”-type response, which allows the detection of hits with decarboxylase-activity. This can be followed by activity order/catalytic efficiency assessments of the hits of decarboxylase activity using the available HPLC methods, which on the other hand, are not suitable for the high-throughput activity screens of large mutagenesis libraries. Moreover, the dysfunctionality of the plate assay or appearance of false positive hits for substrates with significant structural differences to cinnamic acid, e.g. **1p**, **1m** reveals the limitations of the current form of the plate assay, that employs the optimal detection conditions developed for the decarboxylation of cinnamic acid^51^, and highlights ‘substrate-personalization’ perspectives.

Furthermore, is highly notable, that the activity screens/conversion assessments revealed several “hot-spot” active site residues, which upon mutations resulted in FDC variants of improved activity. Interestingly mutation of residue F397 of *Sc*FDC to tyrosine, its homologue residue from *An*FDC, in several cases, such as decarboxylations of **1c**, **1g**, **1l**, **1n** and **1o**, provided increased fluorescent signals (Figures S4, S8, S13, S15, S16, respectively and/or Tables S3, S4, S5) and conversions (Tables S9, S13, S18, S20, S21 respectively and/or Fig. 3). Mono-ethoxy substituted cinnamic acids **1a** and **1b**, transformed by *wild-type Sc*FDC with moderate/good conversions of 62% and 71% (Fig. 3), respectively, were quantitatively decarboxylated by variants I189A/V, suggesting a preferable orientation of the *orto*-, *meta*- substituents towards residue I189. In case of *para*-substituted substrates **1c**–**1e**, mutation of residues F397 and I398 improved the enzyme activity, resulting in conversions up to 78% and 93%, respectively, in comparison with the 39% (for **1c**) and 22% (for **1d**) conversions provided by the *wild-type* enzyme (Fig. 3). The sterically more demanding *tert*-butyl substituted derivate **1e** was not transformed either by *wild-type* or single mutants F397V or I398A(V) variants, however double mutant variants F397V/I398A and F397V/I398V provided conversions of 11% and 24%, respectively, revealing an additive effect of the combined mutations. The active site orientation of the substrate’s aromatic substituents, depicted by the biotransformations of mono-substituted derivatives, were also supported by the decarboxylations of the disubstituted substrates **1f**–**1h.** Notable, that substrate analogues **1f** and **1g**, disubstituted in the *meta*, *para*- position of the aromatic ring, resemble the substitution pattern of the natural substrate, ferulic acid. Thus, expectedly *wild-type Sc*FDC transforms all three disubstituted substrates, obtaining high conversions of 66% in case of **1f**, and moderate/low conversions for the dimethoxy-cinnamic acids (14% in case of **1g**, and 33% in case of **1h)**. In accordance with previous reports^2,7^, this result supports that electronic effects also influence the enzyme activity of FDC. However, aiming to find residues, of which mutations lead to improved activities, we continued to focus on the relative activity increment within the variant library towards each individual substrate. Accordingly, the significant improvements in conversion values in case of 3,4-dimethoxy-cinnamic acid **1g** when using mutant variants I189A/V and I398A (increase with 45–52% in comparison with *wild-type—*Fig. 3, Table S13), corresponds with the supposed orientation of the *meta*- and *para*-substituents towards residues I189 and I398, respectively. In case of 2,5-dimethoxy-cinnamic acid **1h**, besides the expected superior activity of I189A and I398A/I189A variants, reflected in an increase of conversion values with 29% and 33%, relative to those obtained with the *wild-type* FDC, variant I330A provided the highest activity increase (c_I330_ = 73%, while c_wt_ = 33%) among the single mutants (Table S14). This suggests, that in case when substituents occupy both *ortho*-, or *meta*-positions from the different sides of the aromatic ring, two active site residues, namely I189 at one side and I398/I330 on the other side of the active site, are involved in the substituent’s accommodation. Notable, that also in case of **1h**, mutation F397Y, similarly to the decarboxylation of **1c**, **1g**, **1l**, **1n** and **1o**, provided activity increase, in this case additional, for the single mutant I189A, leading to variants F397Y/I189A(V) with improved conversions of 79 and 76% (Fig. 3, Table S14). The biotransformations of the 3,4,5-trimethoxy-cinnamic acid **1i** further supports these correlations, while *wild-type* FDC proved to be inactive, double mutant variants, such as I330A(V)/I398A, I398A(V)/I189A or F397V(Y)/I398A, including mutations of residues I398, F397 (for *para*-positioned substituents) or I189, I330 (for *meta*-substituents) provided superior variants, resulting in conversions of 54–83% (Figs. 2, 3, Table S15).

Definitely, substrate accommodation of bi(hetero)-aromatic substrate analogues **1j**–**1p** is less predictable based on the active site model (Fig. 1), therefore the mutation-activity increase correlation, revealed by the activity measurements, is even more valuable for the structure-guided protein engineering of FDC. Despite that heteroaromatic, bicyclic derivatives **1j** and **1k** show a different structural architecture, with functionalization occurring in the 3’ and 2’ positions of the heteroaryl rings, similar mutations of residues I189, I330 provide variants I189A(V) and I330A, with superior conversions of ~ 90% (**1j**), 45–63% (**1k**) to the moderate conversions of 29% (**1j**) and 39% (**1k**) obtained by the *wild-type* FDC (Table S16, S17, respectively and Fig. 3). Regarding the transformations of bulkier, differently connected biarylic substrate analogues **1l**–**1o**, *wild-type Sc*FDC showed low (12% and 23% conversions for **1l** and **1m**, respectively—Tables S18, S19) or no activity (**1n**, **1o**—Tables S20, S21). Besides the expected beneficial effect of individual or combined mutations of F397A and I398A(V), providing superior variants with good to moderate conversions, mutant variant F397Y also resulted in improved 41.5% and 65.3% conversions for **1n** and **1o**, respectively. Generally, in case of these bulky substrates **1l**–**1o**, mutations of similar hot-spot residues (I189, Q192, F397, I398) provided single/double mutant variants of high activity, leading to conversions between 37 and 81% for compounds **1m**–**o** (Fig. 3, Tables S19–S21).

Accordingly, the activity screens of the FDC variant library towards the substrate panel provided a comprehensive active site map, which correlates the substitution pattern of the substrate’s aromatic moiety with its specific active site positioning, strengthening the rational design-based FDC engineering.

### Computational studies

Based on previous reports^1,7,9^ and ligand bound *An*FDC crystal structures (PDB ID: 4ZA7, 4ZA8, 4ZAB), the proper binding of the substrates implies several requirements. Among them, the location of the α − β double bond of the substrate should be in the proximity of the C1’ and C4a atoms of the cofactor, necessary for the 1,3-dipolar cycloaddition mechanism (Fig. 1 and Fig. S23). Furthermore, in *Sc*FDC, R175 and E285 interact with the carboxyl group of the substrate, while E285 acts also as acid–base in the reaction mechanism, while E280, tunes the pKa of R175 and in turn E285 (Fig. S23)^22^. Accordingly, the reaction rates are influenced by multiple substrate-related factors, such as inductive effects of substituents, presence of extended conjugation, substrate orientation related to the prFMN and within the catalytic site, that is influenced by both the size and planarity of the substrate. Considering these factors and using our previously validated molecular docking method^7^, we attempted to gain molecular level insights on the beneficial effect of the mutations on enzyme activities.

Generally, the obtained computational results were in good agreement with the experimentally observed activity enhancements, revealing proper substrate orientations within the catalytic site of the best performing mutant variants. In the following, representative cases are presented, supporting that in contrast to the *wild-type Sc*FDC, appropriate mutations relieved the steric hindrance between the substrate and the side chain of the corresponding active site residues. Substrate positioning of representative compounds **1e**, **1i**, **1m**, **1o** within the active site of the corresponding best performing FDC variants is in good agreement with the optimized model of the cinnamic acid bound into the *wild-type Sc*FDC1 (Fig. S24).

**Figure 4:**
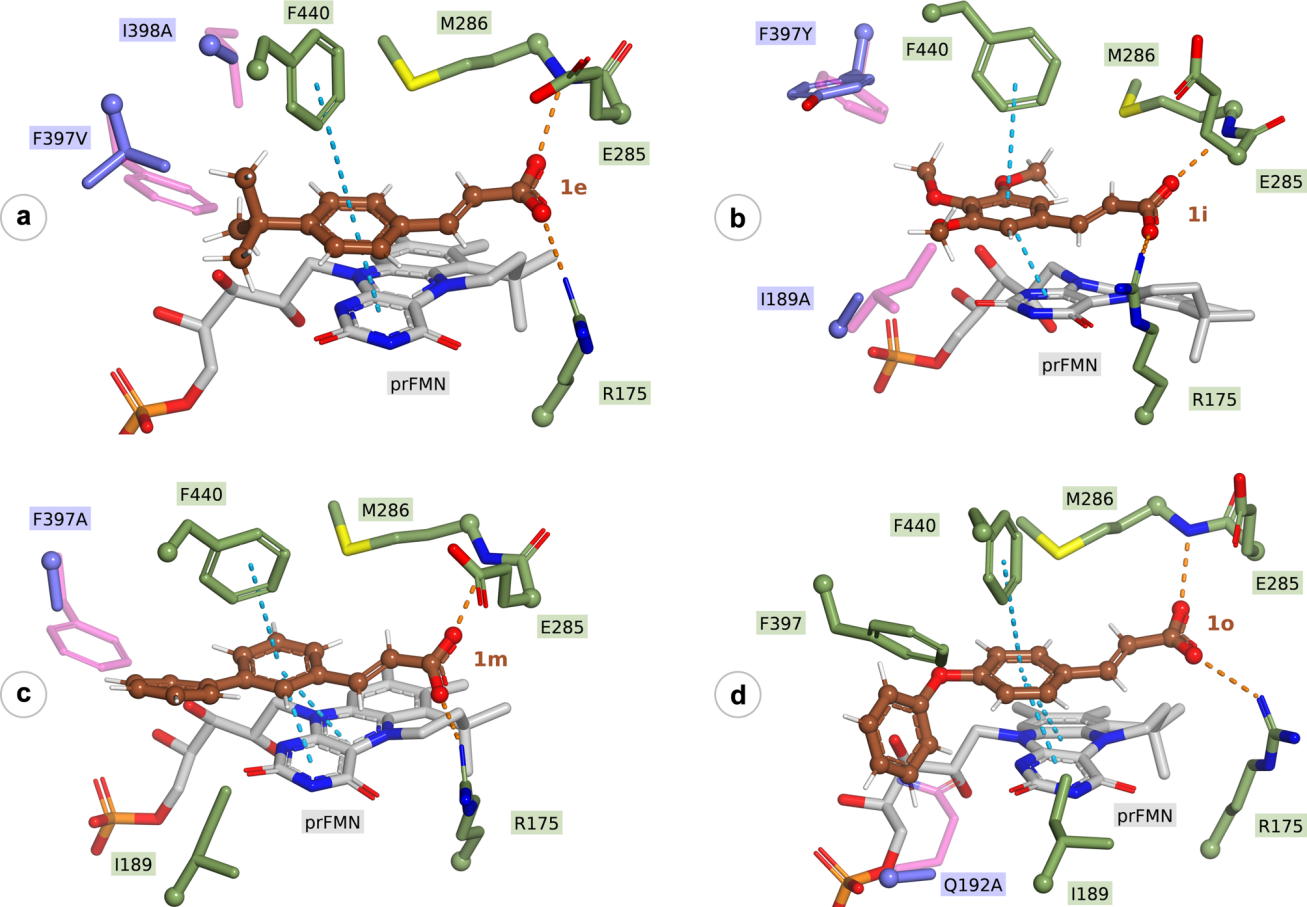
In each figure the side chains of the preserved active site residues of *Sc*FDC1 (green) and the mutant residues (blue) overlaid with their original counterparts from the *wild-type* enzyme (magenta) are represented as stick models. The substrates **1e**, **1i**, **1m**, **1o** are colored in brown within Fig. 4a–d, respectively. Hydrogen bonds between the carboxyl group of the substrate and the backbone nitrogen atom of residue M286 and side chain of residue R175 are indicated as orange dashed lines, whereas the blue dash corresponds to the pi–pi interactions of the substrate’s aromatic ring and residue F440 and the prFMN cofactor, respectively. All these interactions were considered within the selection process of the proper binding state, required for the 1,3-cycloaddition mechanism. *Softwares used for the preparation of images are listed in Supporting information, Section 1*.

In the case of *p*-(*tert*-butyl)cinnamic acid **1e**, converted only by variants F397V/I398A and F397V/I398V (Fig. 3), proper binding orientations of **1e** have been obtained within the catalytic site of both variants (Fig. 4a), while within the *wild-type Sc*FDC no active substrate-binding state could be observed. The inactivity of the *wt*-*Sc*FDC can be attributed to the steric clash between the *tert-*butyl group of substrate **1e** and the aromatic ring of F397, shown in magenta in (Fig. 4a).

In the case of 3,4,5-trimethoxy-cinnamic acid **1i**, the side chains of residues I330 and I189 impede the approach of the substrate to the binding site. The atomic overlap between one of the *meta*-positioned methoxy group of the substrate and the side chain of I189 (shown in magenta) can be observed in Fig. 4b, where the optimized model of the best performing F397Y/I189A variant is represented.

For bulky substrate **1m**, within the best performing variants, mutation F397A provides space for the biaryl ring system, allowing the proper orientation of the double bond for the 1,3-cycloadditions (Fig. 4c). The mutation relieved the steric hindrance between the two aromatic rings of the F397 side chain and substrate **1m**, which can be noticed in Fig. 4c. It is noteworthy that this conformation of the substrate corresponds to the ground state geometry, which could explain the improved conversions observed using variant F397A (c = 79%) with respect to the *wild-type* enzyme (c = 23%).

In case of the similarly bulky substrate **1o**, the etheric group bends the two aromatic rings relatively to each other, resulting in a partial overlap between the *p*-phenoxy substituent group and residue Q192, explaining the enhanced activity of variant Q192A (Fig. 4d). Unfortunately, computational results didn’t provide molecular level evidence for the improved activity of variant F397Y.

The strong correlation of the experimental and computational data suggests that the employed molecular model is suitable also for *in-silico* activity predictions for *Sc*FDC, providing a computational tool for the rational engineering of FDC. Notable, that during the preparation of our manuscript in silico activity predictions were also reported in tailoring *An*FDC and *Sc*FDC for the 1,3-butadiene synthesis^37^.

## Conclusions

Within our study a fluorescent plate assay suitable for the high-throughput (HT) activity screens for ferulic acid decarboxylases (FDCs), has been developed and validated through the activity screens of a mutant library rationally designed towards a 16-membered aromatic substrate panel. Identification of mutant variants of enhanced activities provided correlation between the substitution pattern of substrates with their specific active site accommodation. The computational data supported the experimentally observed activity enhancements and revealed proper substrate orientations within the catalytic site of the best performing mutant variants and supported our molecular model for in silico FDC-activity predictions. Accordingly, the computational activity predictions, the developed HT-activity assay and the identified substrate specificity modulator residues provide a powerful toolbox for the directed evolution or rational designed based protein engineering of FDCs.

## Experimental part

### Materials and methods

See detailed description in *Supporting Information, Chapter 1 and Chapter 2.*

### Mutant library generation

The FDC variant library was obtained through site-directed mutagenesis as described in *Supporting information, Chapter 5*.

### Substrate panel generation

The tested substrate library was obtained through the Knoevenagel-Doebner reaction using the corresponding aromatic aldehydes as starting material. For the detailed description of the procedures, obtained yields and NMR data see *Supporting information, Chapter 3.*

### Initial activity screens with cell-plate assay

For all assay plate, whole-cells of *E. coli* Rosetta (DE3) pLysS were used as expression hosts, harboring the pCDF-Duet1 plasmid carrying the genes of *Scfdc1* and *Scpad1* (*for detailed description of the plasmid construction, molecular cloning see ESI, Chapter 4*).

2 µL of cell suspensions harboring the plasmids of each FDC variant were transferred (pipetted) onto LB-agar Petri plates containing chloramphenicol (34.0 µg/mL), followed by overnight incubation at 37 °C. The colonies grown on the agar plate were transferred on a PVDF membrane, pre-treated by washing with methanol and phosphate buffer (100 mM NaH_2_PO_4_ pH 7.0). For successful colony transfer the membrane was left for 20 min on the plate. Further, the membrane was transferred to an induction plate (LB-agar with 1 mM IPTG and 34.0 µg/mL chloramphenicol) and incubated for 8 h at 30 °C. Cell permeabilization was performed by placing the membrane under chloroform vapours for 45 s using a desiccator, followed by dialysis on 0.4% agarose plate in phosphate buffer (100 mM NaH_2_PO_4_ pH 7.0) and storage at 4 °C, overnight.

The reaction medium plate was prepared by dissolving substrates **1a**–**p** at 1 mM final concentration in 1% agarose gel, followed by the incubation of the membrane on the reaction plate at 37 °C for 4 h. For the fluorescent detection of the colonies with FDC activity, the membrane was placed on a filter paper moistened with a solution of 100 M tetrazole in phosphate buffer (100 mM NaH_2_PO_4_, pH 7.0) and incubated in dark for 1 h at 37 °C, followed by UV-irradiation at 302 nm for 1 min. The detection of signal intensities provided by the colonies of the assay plate was performed by ChemiDoc™ Imaging System, using a corresponding UV filter, allowing detection of specific emissions at > 360 nm wavelengths. The obtained images were analyzed by the Image Lab 5.2.1 software, selecting an area of 2.8 mm^2^ from each spot corresponding to the different colonies, for which the background given by the negative control colony has been decreased from the mean values of all pixels inside the boundary volume. The obtained maximum signal intensity value being considered as 100% relative enzyme activity of the other signal intensities provided the corresponding relative activities. All assay-plates have been performed in duplicates, and in all plates the negative controls were represented by the *E. coli* host cells, harbouring the empty pCDF-DUET1 vector.

### Analytical scale biotransformations

#### Culture preparation

Cultures of *E. coli* BL21(DE3) cells were prepared using Luria–Bertani (LB) medium supplemented with chloramphenicol and streptomycin, that was inoculated with 1–2 v/v % of overnight culture. Following incubation at 37 °C, 220 rpm, the cultures were induced with IPTG (at a final concentration of 0.2 mM) at OD_600_ ~ 0.6, followed by incubation at 25 °C, 220 rpm until to a cell density of OD_600_ ~ 2. The cells were harvested via centrifugation and immediately used in biotransformations.

#### Analytical scale FDC mediated decarboxylations

Stock solutions of each substrate in DMSO (50 mM) were diluted to 2 mM or to 1 mM in the case of substrates of low solubility, **2k**–**p**, with phosphate buffer (100 mM NaH_2_PO_4_, pH 7.0). The freshly prepared, induced *E. coli* cells harboring *fdc1* genes, were resuspended in the reaction solution to a final OD_600_ of 2, followed by incubation of the reaction mixtures at 35 °C, 700 rpm, for 16 h.

#### RP-HPLC monitoring

After 16 h of reaction time, the entire reaction mass was subjected to cellular lysis through sonication, followed by the removal of the cellular pellet through centrifugation at 13,400 rpm, 12,000 g, for 10 min. The cellular pellet was extracted with 500 µL MeOH, which was combined with the supernatant of the previous centrifugation step. 100 µL of the combined solution was diluted with 100 µL of a solution containing 100 mM NaH_2_PO_4_, pH 7.0, acetonitrile, and benzalacetophenone, used as an internal standard (please refer to *Supporting information, Chapter 7., HPLC methods* for the exact composition of this solution). All HPLC analyses were performed at 25 °C using a Phenomenex Kinetex NX-C18 150 × 4.5 mm column, a mobile phase of 30% H_2_O (0.1% v/v TFA) and 70% ACN (0.1% v/v TFA), a flow-rate of 1 mL/min, injecting 5 µL of the previously obtained samples. Conversion values were determined by monitoring the depletion of the substrate concentration, using benzalacetophenone as an internal standard (for detailed description of conversion determination and relative response factors see *Supporting Information, Chapter 7*).

### Molecular docking

The molecular docking calculations were carried out by the Autodock Vina software^53^, using flexible-ligand and rigid-receptor docking. The docking parameters were modified to ensure that among the resulting poses the one with proper binding orientation of the substrate (as defined previously in *Section 2.4*) could be identified. Accordingly, a longer search was employed by setting the exhaustiveness of the search to 100, whereas the energy range between the best and worst binding pose was adjusted to 10 kcal/mol. The dimension of the search space was defined by the binding site residues highlighted in Fig. 1 and the prFMN cofactor, based on the crystal structure of *Sc*FDC1 (PDB: 4ZAC)^1^, therefore a cubic grid box with the size of 18 Å × 18 Å × 18 Å was employed as it can be seen in Figure S25.

The ground state geometries of the substrates were obtained by means of density functional theory. All quantum chemical calculations were performed by Gaussian 09^54^ employing the B3LYP density functional with the 6-31G(d,p) basis set, in a water solvated environment using the Polarizable Continuum Model (PCM)^55^.

The crystallographic structure of *Sc*FDC1 was retrieved from Protein Data Bank entry 4ZAC^1^. The inactive conformation of residue E285 was altered according to those observed in the ligand bound *An*FDC1 crystal structures. The selected docking results were submitted for minimization using the YASARA web server^56^.

## Supplementary Information

Supplementary Information.
