# Supplementary material for: Toolbox for the structure-guided evolution of ferulic acid decarboxylase (FDC)
Source: Sci Rep. 2022 Mar 1;12:3347. doi: 10.1038/s41598-022-07110-w (PMC8888657; doi:10.1038/s41598-022-07110-w)
Supplement: Supplementary file 1 — Supplementary Information. [file 41598_2022_7110_MOESM1_ESM.pdf]

Supporting Information

Table of Contents

1. Materials 2

2. Instrumentation 2

3. Chemical synthesis of substrates **3p**, **3n** and **1a-p** 3

4. Molecular cloning 7

5. Site-directed mutagenesis used for mutant library generation 8

6. Agar-plate FDC1-activity assay 10

7. HPLC methods 23

8. Molecular modeling 37

9. References 39

1. Materials

The commercial chemicals and solvents used in the chemical synthesis of the substrates were purchased were products of Sigma-Aldrich (St. Louis, MO, USA) and/or Alfa-Aesar (Haverhill, MA, USA). LB medium was purchased from Liofilchem (Roseto, Italy), while protease inhibitor cocktail was obtained from Hoffman La-Roche (Basel, Switzerland) and IPTG, Phusion Hot Start DNA Polymerase, dNTPs, DpnI, XhoI, Bpu1102I, agarose from Thermo Fischer Scientific (Waltham, MA, USA).

The primers used for the mutagenesis were purchased from Invitrogen (Waltham, MA, USA). Plasmid extraction kits and other kits for molecular cloning were purchased from Zymo Research (Irvine, CA, USA).

The following softwares were used for the preparation of figures (**Fig. 1-4**, **Fig. S33-S35** and **Schemes S1, S2**): MarvinSketch version 2.10 for the drawings of chemical structures, synthetic routes (**Fig. 1-3, Scheme S1, S2**), Origin PRO 2016 for tables or diagrams from **Fig. 2** an **Fig. 4**; PyMOL 2.5 for the preparation of figures showing protein structures (**Fig. 1b**, **1c**, **Fig. 4.**), SnapGene 5.2.2 for **Fig S1a**, while for the assembly of multiple image files within **Fig. 2** and **Fig. 3** GIMP photo editing software.

2. Instrumentation

The ^{1}H and ^{13}C NMR spectra were obtained with Bruker (Billerica, MA, USA) Avance spectrometers operating at 400 MHz and 101 MHz / 600 MHz and 151 MHz, respectively. Spectra were recorded at 25 °C in $CDCl_3$, D_2O, MeOD, DMSO. ^{1}H and ^{13}C NMR spectra were referenced internally to the solvent signal.

MS spectra and LC-MS analysis were recorded on Agilent 6410 Triple Quadrupole LC/MS mass spectrometry system. LC-MS measurements of **1a-p** were performed using Phenomenex Kinetex 2.6 μm C18 100Å, 50×2.1 mm column, acetonitrile 70%, water (0.1% HCOOH) 30% as mobile phase at 0.3 ml/min flow rate. The MS detector was operated in positive electrospray ionization mode, with source temperature of 35 °C, capillary voltage 4000 V, fragmentor 120 V and with MS2 Scan mode, at least +/- 50 amu around molecular ion.

High performance liquid chromatography (HPLC) analyses were conducted with an Agilent (Santa Clara, CA, USA) 1200, 1260 and 1100 systems. Thin layer chromatography (TLC) was carried out using Merck Kieselgel 60 F254 sheets. Spots were visualized by treatment with 5% ethanolic phosphomolybdic acid or ninhydrin solution and heating. Preparative chromatographic separations were performed using column chromatography on Merck Kieselgel 60 (63-200 μm). The HPLC monitoring of the enzymatic reactions, the determination of conversion values by HPLC was performed using the developed analytical methods (**see ESI, section 7.**).

Analytical scale enzymatic decarboxylations were performed with Heidolph (Schwabach, Germany) Titramax 1100 equipped with incubator module.

For the PCR reactions Mastercycler S from Eppendorf (Hamburg, Germany) was used, while DNA concentration measurements were performed with the NanoQuant plate of the Tecan 10M microplate reader. Gene sequencing services were performed through Biomi (Gödöllő, Hungary).

Within the plate-assays the UV-irradiation at 302 nm was performed using an UVP Transilluminator from Analytik Jena (Jena, Germany), while for the detection of fluorescence signals a ChemiDoc™ Touch Imaging System from Bio-RAD (Hercules, CA, USA) have been employed.

3. Chemical synthesis of substrates 3p, 3n and 1a-p

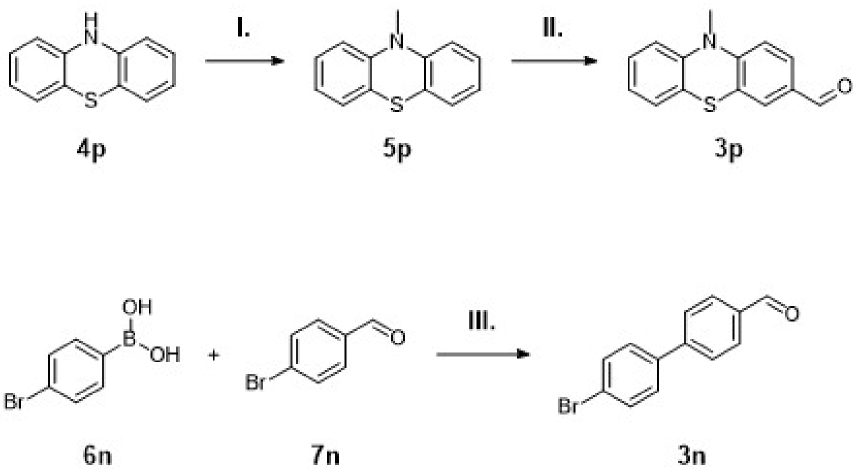

I. NaH, DMF, r.t.; II. $POCl_3$, DMF, 95°C;
III. $Pd(PPh_3)_4$, Na_2CO_3 2M, Toluene/EtOH, 90°C, 5-6h

Scheme 1. Synthetic route for compounds **3p**, **3n.**

I. *Synthesis of 10-methyl-10H-phenothiazine* **5p**

To a stirred solution of NaH (1.45 g, 30.2 mmol, 55% suspension in mineral oil) in dry DMF (30 mL) under argon atmosphere for 20 minutes 10*H*-phenothiazine **4p** (4 g, 20.1 mmol) dissolved in dry DMF (15 mL) was added at 5-10 °C temperature. The solution thus obtained was stirred for 30 min. Then MeI (4.28 g, 30.2 mmol) dissolved in dry DMF (3 mL) was added to the reaction mixture keeping the reaction temperature between 0-10 °C. The raction mixture was stirred at room temperature overnight. The solution was poured on a water ice mixture (200 mL) and the formed precipitate was filtered off and dried under reduced pressure. The product was purified by silica gel column chromatography using *n*-hexane:EtOAc 8:2 as eluent, leaving the pure **5p** (yield: 45-55%).

II. *Synthesis of 10-methyl-10H-phenothiazine-3-carbaldehyde* **3p**

The 10-methyl-10*H*-phenothiazine **5p** (2.53 g, 11.9 mmol) was dissolved in DMF (2.4 mL) and $POCl_3$ (2.38 g, 1.5 mL, 15.5 mmol) was added dropwise. The reaction mixture was stirred at 95 °C overnight. The solution was poured on a water ice mixture (200 mL) and 10% NaOH was added to set pH 7. Then the solution was extracted with CH_2Cl_2 (3 x 20 mL), dried and purified by silica gel column chromatography using CH_2Cl_2 as eluent, leaving the pure **3p** (yield: 80-90%).

III. *Synthesis of* 4'-bromo-[1,1'-biphenyl]-4-carbaldehyde **3n**

4-bromobenzaldehyde (**7n**, 0.3 g, 1.6 mmol) and (4-bromophenyl)boronic acid (**6n**, 0.48 g, 2.4 mmol) were dissolved in a mixture of toluene:EtOH 1:1 (7-7 mL) under argon atmosphere. Then 2M Na_2CO_3 (0.4 g, 3.2 mmol) and $Pd(PPh_3)_4$ (0.18 g, 0.16 mmol) was added to the reaction mixture. The reaction mixture was then stirred at 90 °C for 5-6 h. The solution was then cooled down to room temperature and extracted with EtOAc (3 x 20 mL), dried and the solvent was evaporated under vacuum. The product was purified by silica gel column chromatography using CH_2Cl_2 as eluent, leaving the pure **3n** (yield: 50-60%).

IV. *Synthesis of cinnamic acid derivatives* **1a-p**

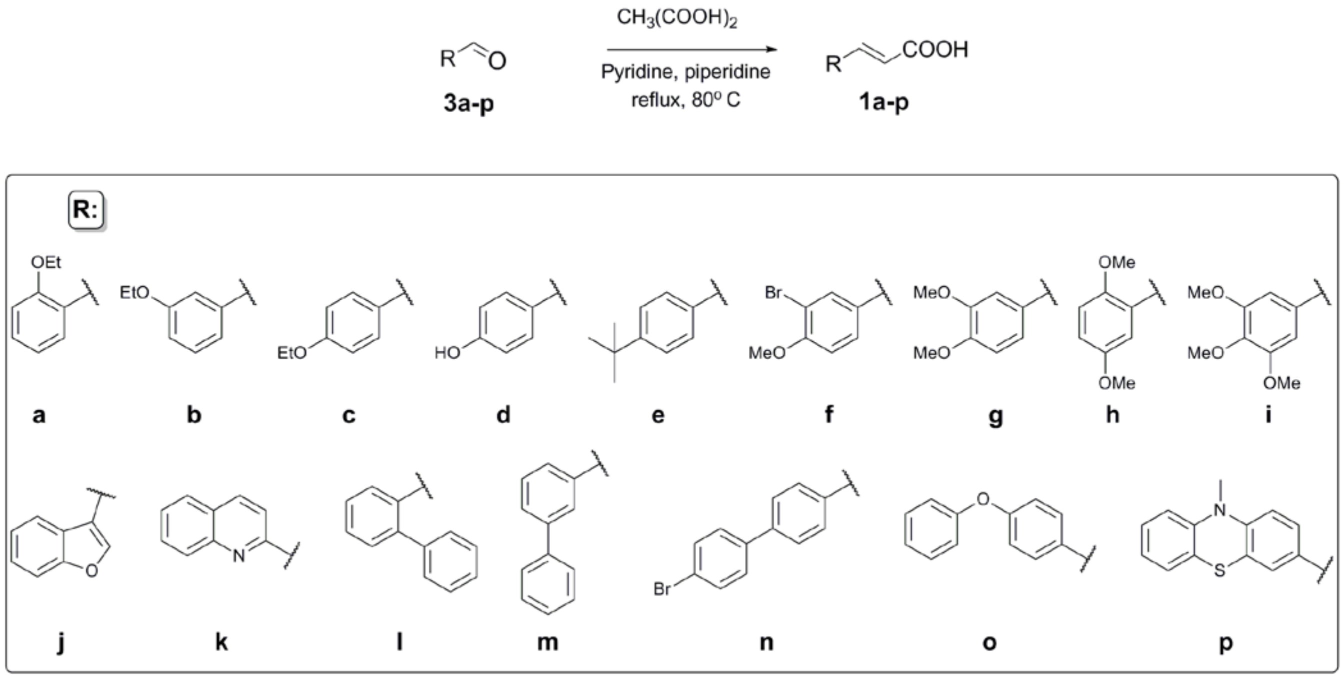

Scheme 2. Synthesis of cinnamic acid derivatives **1a-p.**

The aldehyde **3a-p** (1 g, 5.75-10 mmol) and malonic acid (2 equiv.) were dissolved in pyridine (20 mL) and piperidine was added (0.3 mL). The mixture was heated under reflux for 6 h. The solution was then cooled in an ice bath and poured in aq. HCl (50 mL, 3 M). The white solid that precipitated was filtered and washed with water (3 × 10 mL), diethyl-ether (20 mL) and finally dried to afford pure acrylic acids **1a-p** (yields: 78-82%).

(E)-3-(2-ethoxyphenyl)acrylic acid **1a***:*

^{1}H NMR (600 MHz, Methanol-d_4) δ 7.89 (d, J = 16.1 Hz, 1H), 7.45 (d, J = 7.7 Hz, 1H), 7.24 (t, J = 7.9 Hz, 1H), 6.89 (d, J = 8.3 Hz, 1H), 6.84 (t, J = 7.5 Hz, 1H), 6.43 (d, J = 16.1 Hz, 1H), 4.00 (q, J = 6.9 Hz, 2H), 1.35 (t, J = 6.9 Hz, 3H). ^{13}C NMR (151 MHz, MeOD) δ 171.00, 159.10, 141.85, 132.83, 129.80, 124.30, 121.69, 119.25, 113.31, 65.12, 49.43, 49.28, 49.14, 49.00, 48.86, 48.72, 48.57, 15.09.

(E)-3-(3-ethoxyphenyl)acrylic acid **1b***:*

^{1}H NMR (600 MHz, Methanol-d_4) δ 7.56 (d, J = 16.0 Hz, 1H), 7.23 (t, J = 7.9 Hz, 1H), 7.08 (d, J = 7.8 Hz, 1H), 7.04 (s, 1H), 6.89 (d, J = 8.2 Hz, 1H), 6.39 (d, J = 16.0 Hz, 1H), 3.99 (q, J = 7.0 Hz, 2H), 1.33 (t, J = 7.0 Hz, 3H). ^{13}C NMR (151 MHz, MeOD) δ 170.32, 160.84, 146.35, 137.13, 130.97, 121.61, 119.49, 117.78, 114.62, 64.58, 49.43, 49.28, 49.14, 49.11, 49.00, 48.86, 48.72, 48.57, 15.12.

(E)-3-(4-ethoxyphenyl)acrylic acid **1c***:*

^{1}H NMR (600 MHz, Methanol-d_4) δ 7.58 (d, J = 16.0 Hz, 1H), 7.49 (s, 2H), 6.90 (s, 2H), 6.29 (d, J = 16.0 Hz, 1H), 4.03 (s, 2H), 1.35 (s, 3H). ^{13}C NMR (151 MHz, MeOD) δ 170.82, 162.41, 146.27, 130.91, 128.26, 116.43, 115.88, 64.67, 49.43, 49.28, 49.14, 49.03, 49.00, 48.86, 48.72, 48.57, 15.05.

(E)-3-(4-hydroxyphenyl)acrylic acid **1d***:*

^{1}H NMR (400 MHz, Methanol-d_4) δ 7.51 (d, J = 15.9 Hz, 1H), 7.36 (d, J = 8.7 Hz, 2H), 6.71 (d, J = 8.6 Hz, 2H), 6.19 (d, J = 15.9 Hz, 1H). ^{13}C NMR (101 MHz, MeOD) δ 171.04, 161.16, 146.66, 131.09, 127.19, 116.78, 115.55, 49.64, 49.43, 49.21, 49.00, 48.79, 48.57, 48.36.

(E)-3-(4-(tert-butyl)phenyl)acrylic acid **1e***:*

^{1}H NMR (600 MHz, Methanol-d_4) δ 7.59 (d, J = 15.9 Hz, 1H), 7.42 (d, J = 35.0 Hz, 4H), 6.38 (d, J = 16.0 Hz, 1H), 1.26 (s, 9H). ^{13}C NMR (151 MHz, MeOD) δ 170.54, 155.06, 146.28, 133.03, 129.06, 126.94, 118.37, 49.43, 49.28, 49.14, 49.00, 48.86, 48.72, 48.57, 35.70, 31.55.

(E)-3-(3-bromo-4-methoxyphenyl)acrylic acid **1f***:*

^{1}H NMR (400 MHz, DMSO-d_6) δ 7.96 (s, 1H), 7.79 – 7.60 (m, 1H), 7.51 (d, J = 15.7 Hz, 1H), 7.14 (s, 1H), 6.46 (d, J = 15.4 Hz, 1H), 3.88 (s, 3H). ^{13}C NMR (101 MHz, DMSO) δ 167.67, 156.79, 142.35, 132.56, 129.42, 128.45, 118.15, 112.80, 111.23, 56.52, 40.15, 39.94, 39.73, 39.52, 39.31, 39.10, 38.89.

(E)-3-(3,4-dimethoxyphenyl)acrylic acid **1g***:*

^{1}H NMR (600 MHz, Methanol-d_4) δ 7.40 (d, J = 15.9 Hz, 1H), 6.99 (s, 1H), 6.95 (d, J = 8.2 Hz, 1H), 6.76 (d, J = 8.2 Hz, 1H), 6.15 (d, J = 15.9 Hz, 1H), 3.65 (d, J = 2.8 Hz, 6H). ^{13}C NMR (151 MHz, MeOD) δ 170.76, 152.69, 150.70, 146.47, 128.81, 123.89, 116.84, 112.56, 111.40, 56.47, 56.40, 49.43, 49.28, 49.14, 49.00, 48.86, 48.72, 48.57.

(E)-3-(2,5-dimethoxyphenyl)acrylic acid **1h***:*

^{1}H NMR (400 MHz, Methanol-d_4) δ 7.80 (d, *J* = 16.2 Hz, 1H), 6.96 (s, 1H), 6.81 (d, *J* = 1.7 Hz, 2H), 6.34 (d, *J* = 16.1 Hz, 1H), 3.69 (s, 3H), 3.62 (s, 3H). ^{13}C NMR (101 MHz, MeOD) δ 170.85, 155.05, 154.12, 141.37, 124.81, 119.55, 118.37, 114.02, 113.67, 56.55, 56.15, 49.64, 49.43, 49.21, 49.00, 48.79, 48.57, 48.36.

(E)-3-(3,4,5-trimethoxyphenyl)acrylic acid **1i***:*

^{1}H NMR (600 MHz, Methanol-d_4) δ 7.55 (d, *J* = 15.9 Hz, 1H), 6.83 (s, 2H), 6.37 (d, *J* = 15.9 Hz, 1H), 3.81 (s, 6H), 3.75 (s, 3H); ^{13}C NMR (151 MHz, MeOD) δ 170.40, 154.70, 146.37, 141.05, 131.60, 118.60, 106.54, 61.13, 56.60, 49.43, 49.28, 49.14, 49.00, 48.86, 48.72, 48.57.

(E)-3-(benzofuran-3-yl)acrylic acid **1j***:*

^{1}H NMR (600 MHz, CD_3OD) δ: 8.02 (s, 1H), 7.75 (d, *J* = 7.5 Hz, 1H), 7.70 (d, *J* = 16.1 Hz, 1H), 7.42 (d, *J* = 8.0 Hz, 1H), 7.25 (dd, *J* = 11.8, 7.7 Hz, 2H), 6.47 (d, *J* = 16.1 Hz, 1H); ^{13}C NMR (151 MHz, CD_3OD) δ: 169.22, 157.55, 150.24, 136.18, 126.53, 125.81, 124.98, 121.95, 118.94, 118.55, 112.81, 49.43, 49.28, 49.14, 49.00, 48.86, 48.72, 48.57.

(E)-3-(quinolin-2-yl)acrylic acid **1k***:*

^{1}H NMR (400 MHz, DMSO-d_6) δ: 9.41 – 7.25 (m, 7H), 7.02 (d, *J* = 16.0 Hz, 1H); ^{13}C NMR (101 MHz, DMSO) δ: 167.23, 153.12, 147.49, 143.50, 137.09, 130.24, 129.20, 127.90, 127.79, 127.40, 124.60, 120.87, 40.15, 39.94, 39.73, 39.52, 39.31, 39.10, 38.89.

(E)-3-([1,1'-biphenyl]-2-yl)acrylic acid **1l***:*

^{1}H NMR (600 MHz, Methanol-d_4) δ 7.74 (d, *J* = 7.8 Hz, 1H), 7.62 (d, *J* = 15.9 Hz, 1H), 7.46 – 7.18 (m, 8H), 6.37 (d, *J* = 15.9 Hz, 1H). ^{13}C NMR (151 MHz, MeOD) δ 170.19, 145.18, 144.38, 141.45, 133.64, 131.53, 131.09, 130.85, 129.40, 128.92, 128.65, 127.87, 120.15, 49.43, 49.28, 49.14, 49.00, 48.86, 48.72, 48.57.

(E)-3-([1,1'-biphenyl]-3-yl)acrylic acid **1m***:*

^{1}H NMR (600 MHz, DMSO-d_6) δ 7.98 (s, 1H), 7.71 (td, *J* = 16.9, 16.0, 7.6 Hz, 5H), 7.59 – 7.33 (m, 4H), 6.67 (d, *J* = 16.0 Hz, 1H). ^{13}C NMR (151 MHz, DMSO) δ 167.67, 143.92, 140.80, 139.50, 134.97, 129.54, 128.97, 128.48, 127.77, 127.11, 126.89, 126.63, 119.76, 39.93, 39.80, 39.66, 39.52, 39.38, 39.24, 39.10.

(E)-3-(4'-bromo-[1,1'-biphenyl]-4-yl)acrylic acid **1n**:

^{1}H and ^{13}C NMR measurements couldn't be performed because of substrate solubility issues in the tested solvents (DMSO-d6, MeOD-d4 and D_2O/NaOD, MeCN-d3). Therefore, the purity and identity of the compound was determined through LC-MS analysis has been performed on an Agilent 1200 LC system

coupled to an Agilent 6410B triple quadrupole mass spectrometer. The ion source was operated in negative ion mode. The pseudomolecular ion $[M-H]^-$ was observed at m/z 301/303.

(E)-3-(4-phenoxyphenyl)acrylic acid **1o***:*

^{1}H NMR (400 MHz, Methanol-d_4) δ 7.56 (d, J = 16.0 Hz, 1H), 7.49 (d, J = 8.3 Hz, 2H), 7.30 (t, J = 7.8 Hz, 2H), 7.08 (t, J = 7.4 Hz, 1H), 6.92 (dd, J = 26.3, 8.2 Hz, 4H), 6.31 (d, J = 16.0 Hz, 1H). ^{13}C NMR (101 MHz, MeOD) δ 170.50, 161.01, 157.57, 145.62, 131.09, 131.03, 130.60, 125.26, 120.72, 119.33, 117.99, 49.64, 49.43, 49.21, 49.00, 48.79, 48.57, 48.36.

(E)-3-(10-methyL-10H-phenothiazin-3-yl)acrylic acid **1p***:*

^{1}H NMR (400 MHz, DMSO-d_6) δ: 7.73 – 6.65 (m, 8H), 6.43 (s, 1H), 2.50 (s, 3H); ^{13}C NMR (101 MHz, DMSO-d_6) δ: 167.79, 146.82, 144.47, 142.92, 128.70, 128.48, 127.93, 126.88, 126.18, 122.95, 122.49, 121.49, 117.07, 114.94, 114.66, 40.15, 39.94, 39.73, 39.52, 39.31, 39.10, 38.89, 35.36.

4. Molecular cloning

The pCDFDuet-1 vector containing two multiple cloning sites (MCS) was employed for the molecular cloning of both *Sc*FDC and *ScPAD*. The gene of *Sc*FDC, obtained through digestion of pTfdc1Sc plasmid[1] with restriction enzymes *SalI* and *HindIII*, together with *SalI* and *HindIII* digested pCDFDuet-1 vector were extracted from the agarose gel and ligated (1 h incubation at 22 °C) using T4 DNA ligase. 5 µL from the ligation reaction were transformed through heat-shock into *E. coli* XL-1 Blue competent cells, the grown colonies were selected and the presence of the insert (*fdc1* gene) was verified through colony PCR (**Fig. S1b**). The primers used for colony PCR were:

T7_for: 5'AATACGACTCACTATAGGGGAATTG3'

Duet_DOWN1: 5'GATTATGCGGCCGTGTACAA3'.

From one of the positive colonies an overnight culture was grown (5 mL sterile LB supplemented with tetracycline 12.5 µg/mL and streptomycin 30 µg/mL) at 37 °C and 180 rpm and the plasmid fdc1-pCDFDuet-1 was extracted.

Next the synthetic, truncated version of the *tSc*PAD gene[2] was cloned into MCS-2 of the vector already containing the *fdc1* gene in MCS-1 (fdc1-pCDFDuet-1) using restriction sites for *NdeI* and *XhoI*. After transformation through heat-shock of the ligation mixture into *E. coli* XL-1 Blue competent cells, the presence of the second insert (*tPAD* gene) in the grown colonies was tested through colony PCR using the primers: Duet_UP2: 5'TTGTACACGGCCGCATAATC3' and T7_rev: 5'TGCTAGTTATTGCTCAGCGG3'. One of the positive colonies was used to inoculate an overnight

culture (5 mL sterile LB supplemented with tetracycline 12.5 µg/mL and streptomycin 30 µg/mL) at 37 °C and 180 rpm and the plasmid tPAD1-fdc1-pCDFDuet-1 was extracted.

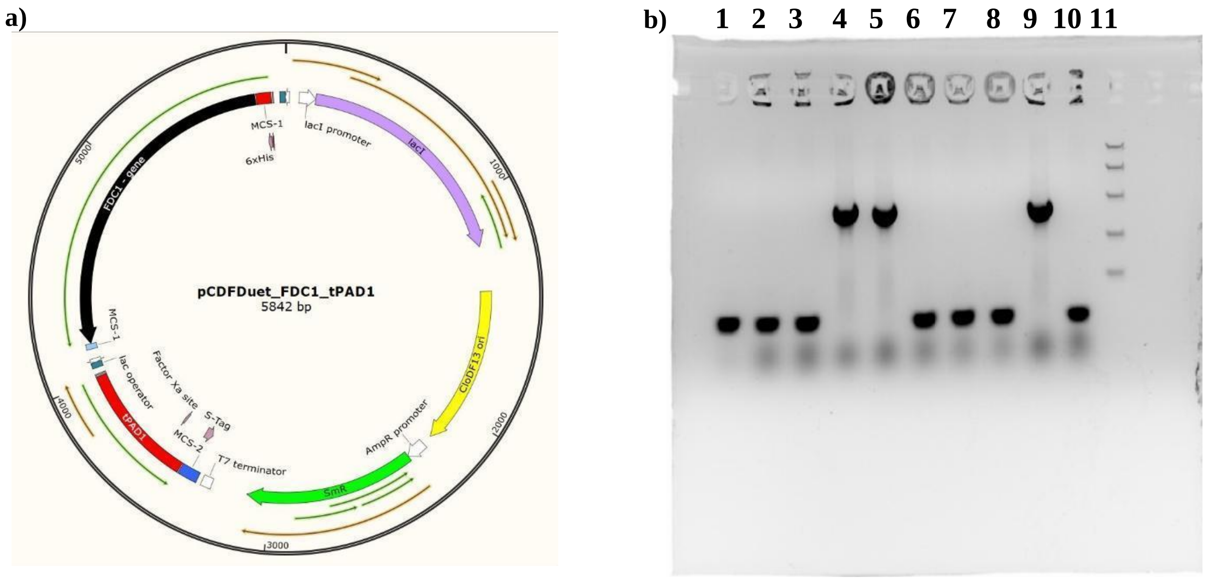

Figure S1. a) Plasmid map for the FDC1/tPAD_pCDFDuet-1 construct and **b)** full-length agarose gel for colony PCR confirming the presence of *fdc1* gene (1756 bp) in pCDFDuet-1 vector in colonies 4, 5 and 9 (**1-10**: colonies, **11**: DNA ladder: 10000, 4000, 2000, 1000, 500 bp).

5. Site-directed mutagenesis used for mutant library generation

FDC1 mutant variants used within activity screens (**Table S2**) were obtained through site-directed mutagenesis using the overlapping PCR method described by Liu and Naismith[3]. The PCR reaction mixtures, with a total volume of 50 µL, contained 10 µL of 5X Phusion HF Buffer, 2-4 ng of template DNA (*Scfdc1* gene cloned in pCDFDuet vector), 2 µM final concentration of the corresponding primer pair (**Table S1**), dNTPs (200 µM) and 2 U of Phusion High-Fidelity DNA polymerase.

The PCR protocol consisted of initial denaturation at 95 °C for 5 minutes, followed by 20 amplification cycles, each of them consisting of denaturation at 95 °C for 5 minutes, annealing at temperature of $T_{m\ no}$ -5 °C for 1 min and extension at 72 °C for 15 minutes. The final annealing step for PCR cycles was finished using $T_{m\ pp}$-5 °C for 1 minute and a final extension step at 72 °C for 30 minutes. 10 µL from each PCR reaction were digested with 5 units of *DpnI* restriction enzyme at 37 °C for 2 h to remove the template DNA, followed by agarose gel electrophoresis to confirm the success of the DNA amplification. 5 µL from the above digested product was transformed into 50-100 µL of *E. coli* XL1-Blue chemically competent cells by heat shock. The transformed cells were spread on a LB-agar (LB) plate containing 25 µg/mL streptomycin and 10 µg/mL tetracycline and incubated at 37 °C for 16 h. Colonies

from each plate have been selected and grown in LB liquid medium, followed by plasmid isolation. The presence of the desired mutations was determined through DNA sequencing carried out by Biomi Ltd. (Gödöllő, Hungary). Once the mutations were confirmed, the corresponding plasmids were transformed by heat-shock into expression host *E. coli* Rossetta (DE3) pLysS, used later within the plate-assays and whole-cell biotransformations.

Table S1. List of the obtained ScFDC mutants and the primers used for site-directed mutagenesis

Entry	Primer name	Sequence (5’-3’) of primers used for mutagenesis	$T_{m\ pp}$ (ºC)	$T_{m\ no}$ (ºC)	$T_{m\ full}$ (ºC)
**1**	I189A	FP: 5’TCTGGTAGCTAAACCACAACATATTAGACAAATTGCTGAC3’	52	62	75
		RP: 5’TGGTTTAGCTACCAGACCAGTGATATGCTTGTCATC3’	52	61	77
**2**	I189V	FP: 5’GTCTGGTAGTTAAACCACAACATATTAGACAAATTGCTGACTCTTG3’	53	61	70
		RP: 5’GTGGTTTAACTACCAGACCAGTGATATGCTTGTCATCTACAAC3’	53	61	71
**3**	Q192N	FP: 5’TTAAACCAAATCATATTAGACAAATTGCTGACTCTTGGG3’	53	61	75
		RP: 5’GTCTAATATGATTTGGTTTAATTACCAGACCAGTGATATGC3’	53	61	73
**4**	Q192S	FP: 5’TTAAACCATCACATATTAGACAAATTGCTGACTCTTGGG3’	54	61	76
		RP: 5’GTCTAATATGTGATGGTTTAATTACCAGACCAGTGATATGC3’	54	61	74
**5**	Q192A	FP: 5’TAAACCAGCACATATTAGACAAATTGCTGACTCTTGG3’	51	61	75
		RP: 5’CTAATATGTGCTGGTTTAATTACCAGACCAGTGATATGC3’	51	61	74
**6**	M286A	FP: 5’GTGAGGCGCATGGATATGTTTTCAAAAGCCAAGGTCATC3’	55	62	74
		RP: 5’CCATGCGCCTCACCAAATGGGCCTTCCAGATGTG3’	55	63	77
**7**	M286V	FP: 5’GGTGAGGTGCATGGATATGTTTTCAAAAGCCAAGGTCATC3’	54	62	73
		RP: 5’CCATGCACCTCACCAAATGGGCCTTCCAGATGTG3’	54	62	77
**8**	I330A	FP: 5’CCTTGGCTGGTTCACTAGTGGCTACTGAGGCC3’	54	61	80
		RP: 5’GAACCAGCCAAGGTATGTGTCTCATCCGTACAAAG3’	54	62	79
**9**	I330V	FP: 5’CCTTGGTTGGTTCACTAGTGGCTACTGAGGCCAAG3’	56	65	80
		RP: 5’GTGAACCAACCAAGGTATGTGTCTCATCCGTACAAAGAC3’	56	64	80
**10**	I398A	FP: 5’GGTTTTGCAGTCCATGAAATAATTTTGGTGGCAGATG3’	52	57	70
		RP: 5’ATGGACTGCAAAACCAACTTTTGTCCTAAAGTAAATATCAC3’	52	57	69
**11**	I398V	FP: 5’GGTTTTGTAGTCCATGAAATAATTTTGGTGGCAGATGATATC3’	52	59	69
		RP: 5’TCATGGACTACAAAACCAACTTTTGTCCTAAAGTAAATATCACC3’	52	59	69
**12**	F397V	FP: 5’GTTGGTGTTATAGTCCATGAAATAATTTTGGTGGCAGATG3’	51	59	69
		RP: 5’TGGACTATAACACCAACTTTTGTCCTAAAGTAAATATCACCTACC3’	51	60	70
**13**	F397A	FP: 5’GTTGGTGCTATAGTCCATGAAATAATTTTGGTGGCAGATG3’	52	60	70
		RP: 5’GGACTATAGCACCAACTTTTGTCCTAAAGTAAATATCACCTACC3’	52	60	70
**14**	F397Y	FP: 5’GTTGGTTATATAGTCCATGAAATAATTTTGGTGGCAGATG3’	51	56	75
		RP: 5’CATGGACTATATAACCAACTTTTGTCCTAAAGTAAATATCACC3’	51	56	72
**15**	F397V/I398A	FP: 5’GGTGTTGCAGTCCATGAAATAATTTTGGTGGCAGATGATATCG3’	56	61	72
		RP: 5’CATGGACTGCAACACCAACTTTTGTCCTAAAGTAAATATCACCTAC 3’	56	61	72
**16**	F397V/I398V	FP: 5’GGTGTTGTAGTCCATGAAATAATTTTGGTGGCAGATGATATC3’	52	59	70
		RP: 5’CATGGACTACAACACCAACTTTTGTCCTAAAGTAAATATCACC3’	52	59	70
**17**	L442V/F440A	FP: 5’CTTCTGCTCCTGTGGCTCCCTTTGTTTCGCAGTC3’	54	60	75
		RP: 5’CCACAGGAGCAGAAGTGACATCATCAAAAGCCATCTG3’	54	60	73
**18**	Duet_DOWN1	- sequencing primer 5’GATTATGCGGCCGTGTACAA3’			
**19**	T7promoter	- sequencing primer 5’GTAATACGACTCACTATAGGG3’			

Table S2. Alignment of active site residues (marked with background) in *Sc*FDC (blue) and *An*FDC (red). Three different active site residues (marked with blue backround) are: P319, F397 and I398 of *Sc*FDC, that corresponds to residues C316, Y394 and T395 in *An*FDC.

	175	187 188 189 190 191 192	283 284 285 286 287	319	326	330	397 398	440 441 442
*Sc*FDC	..R..	..LVIKPQ..	..FGEMH..	..P..	..T..	..I..	..FI..	..FPL..
Uniprot ID: Q03034	|	| | | | |	| | | | |		|	|		| | |
*An*FDC	..R..	..LVIPPQ..	..FGEMH..	..C..	..T..	..I..	..YT..	..FPL..
Uniprot ID: A2QHE5	173	185 186 187 188 189 190	280 281 282 283 284	316	323	328	394 395	437 438 439

6. Agar-plate FDC1-activity assay

For all the experiments, whole-cells of *E. coli* Rosetta (DE3) pLysS were used as expression hosts, harbouring the pCDF-Duet1 plasmid carrying the genes of *fdc1* and *tpad1*.

2 µL of cell suspensions of each FDC variant were transferred (pipetted) onto LB-agar Petri plates containing chloramphenicol (34.0 µg/mL), followed by incubation of plates overnight at 37 °C. The colonies grown on the agar plate were transferred on a PVDF membrane, pre-treated by washing with methanol and 100 mM sodium phosphate buffer, pH=7. For succesful colony transfer the membrane was left for 20 minutes on the plate. Further, the membrane was transferred to an induction agar plate (1 mM IPTG and chloramphenicol) and incubated for 8 h at 30 °C. Cell permeabilization was performed by placing the membrane under chloroform vapours for 45 second using a desiccator, followed by dialysis on 0.4% agarose plate in phosphate buffer (100 mM NaH_2PO_4, pH 7.0) and storage at 4 °C, overnight.

The reaction medium plate was prepared by dissolving substrates **1a-p** at 1 mM final concentration in 1% agarose gel, followed by the incubation of the membrane on the reaction plate at 37 °C for 4 hours. For the fluorescent detection of the colonies with FDC activity, the membrane was placed on a filter paper moistened with a solution of 100 M tetrazole in phosphate buffer (100 mM NaH_2PO_4, pH 7.0) and incubated in dark for 1h at 37 °C, followed by irradiation within a Transilluminator at 302 nm for 1 min. The detection of signal intensities provided by the colonies of the assay plate was performed by ChemiDoc™ Imaging System, using the UV filter within the Gel Green application for nucleic acids. The obtained images were analyzed by the Image Lab 5.2.1 software, selecting an area of 2.8 mm^2 from each spot corresponding to the different colonies, for which the background given by the negative control colony has been decreased from the mean values of all pixels inside the boundary volume. (*E. coli* Rosetta empty expression vector). The obtained maximum signal intensity value being considered as 100% relative enzyme activity of the other signal intensities provided the corresponding relative activities. All assay-plates have been performed in duplicates, and in all plates the negative controls were represented by the *E. coli* host cells, harbouring the empty pCDF-DUET1 vector.

Coloured images of the fluorescent plates, as presented in **Figure 2** (main manuscript) have been obtained by manual photographing of the membrane irradiated on a Transilluminator at 302 nm, showing the blue, fluorescent emission of active colonies. Colony numbering was introduced using GIMP photo editing software, while no other modifications of the image has been performed. The results in Tables S3, S4, S5 present the relative fluorescent signal intensities (RFI) obtained from the corresponding images of the plate assay performed in case of each substrate **1a-p**.

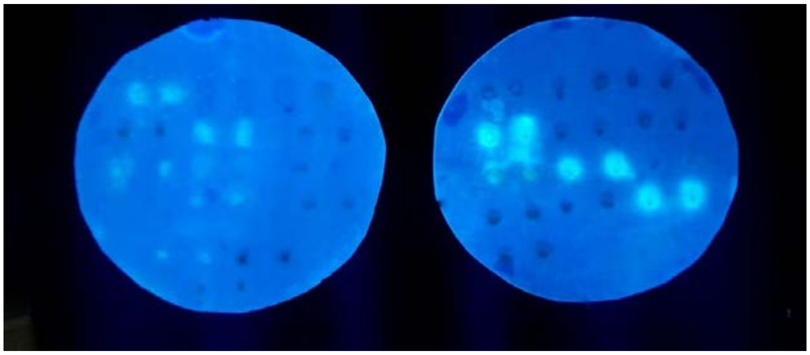

Original, full-length image used within **Figure 2**.

Table S3. Relative fluorescent signal intensities (%) resulted from the plate-assay of the FDC variant library within the decarboxylation of **1a-e**.

**Entry.**	***Sc*FDC**	**Substrates 1a-1e**				
		**1a**	**1b**	**1c**	**1d**	**1e**
1	**wt**	35	61	47	33	0
2	**M1-**I398A	28	75	88	42	0
3	**M2-**F397A	9	84	90	100	0
4	**M3-**M286A	0	0	1	0	0
5	**M4-**M286V	19	28	14	1	0
6	**M5-**F397V/I398A	1	51	77	66	100
7	**M6-**L442V/F440A	0	0	3	0	0
8	**M7-**I330V/I398A	13	34	11	51	0
9	**M8-**Q192A	15	46	33	65	0
10	**M9-**Q192N	5	31	100	80	0
11	**M10-**Q192S	8	41	63	60	0
12	**M11-**I330A	20	21	16	28	0
13	**M12-**I330V	41	42	17	33	0
14	**M13-**I189A	100	100	18	34	0
15	**M14-**I189V	98	66	25	21	0
16	**M15-**F397Y	95	50	28	33	0
17	**M16-**F397V	11	1	0	0	0
18	**M17-**I398V	37	54	29	47	0
19	**M18-**F397V/I398V	9	47	58	42	43
20	**M19-**I330A/I398A	9	43	1	43	0
21	**M20** -I398V/I189A	99	53	21	11	0
22	**M21 -** I398A/I189A	35	46	31	51	0
23	**M22 -** F397Y/I189V	100	48	35	35	0
24	**M23 -** F397Y/I189A	31	47	26	9	0

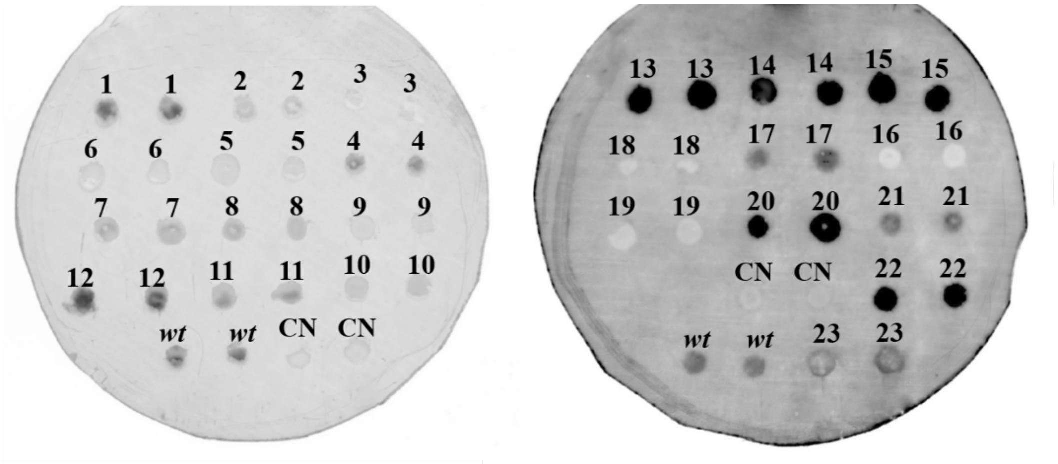

Figure S2. The plate assay showing the fluorescent signals provided by the rationally designed 23 mutant-library of *Sc*FDC (1 to 23) in case of substrate **1a.** CN= negative control: *E. coli* Rosetta (*DE3*), without the plasmid harbouring FDC, *wt*=*wild-type* FDC. (The image was obtained using ChemiDoc™ Imaging System, selecting the UV filter and the Gel Green application for nucleic acids; colony numbering was introduced using GIMP photo editing software)

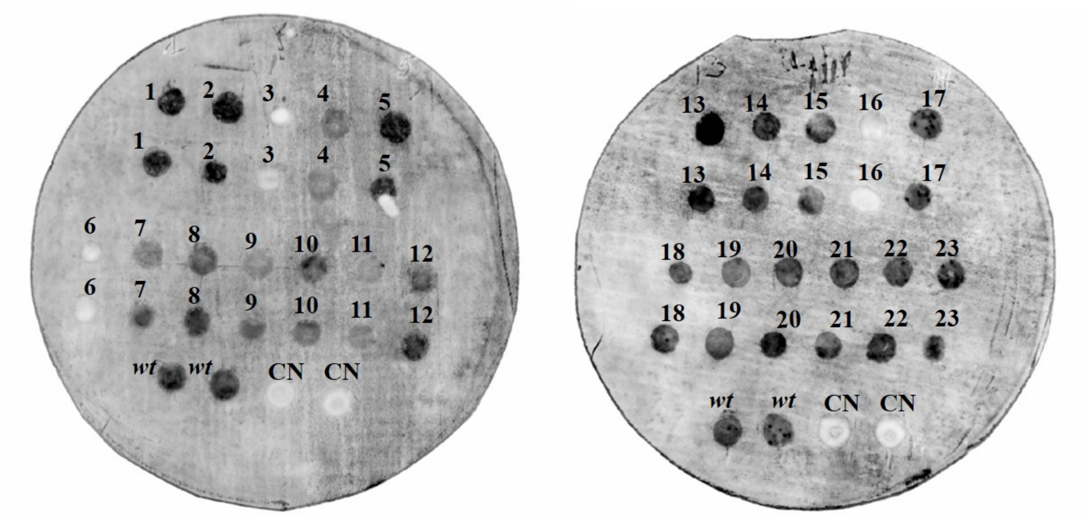

Figure S3. The plate assay showing the fluorescent signals provided by the rationally designed 23 mutant-library of *Sc*FDC (1 to 23) in case of substrate **1b.** CN= negative control: *E. coli* Rosetta (*DE3*), without the plasmid harbouring FDC, *wt*=*wild-type* FDC. (The image was obtained using ChemiDoc™ Imaging System, selecting the UV filter and the Gel Green application for nucleic acids; colony numbering was introduced using GIMP photo editing software)

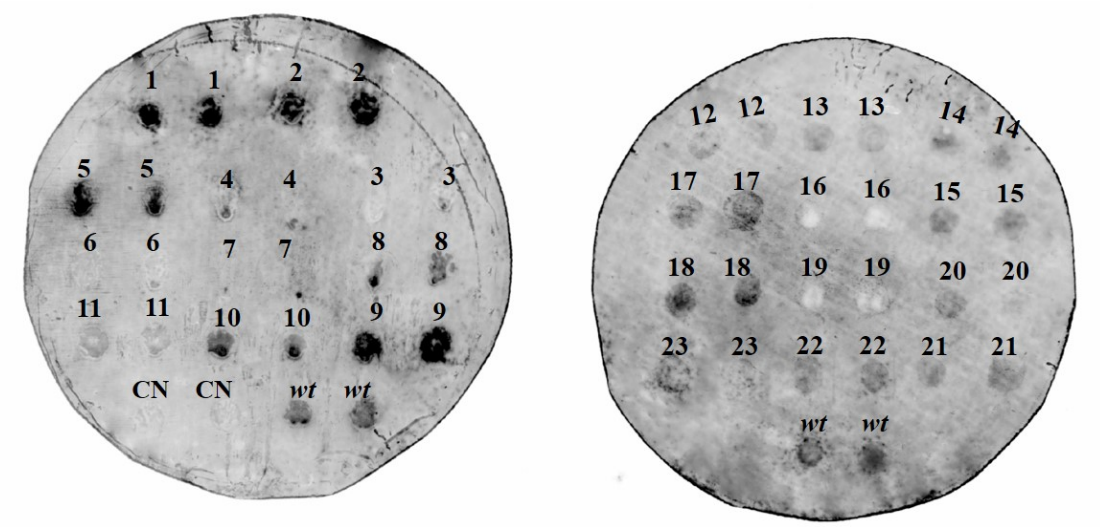

Figure S4. The plate assay showing the fluorescent signals provided by the rationally designed 23 mutant-library of *Sc*FDC (1 to 23) in case of substrate **1c.** CN= negative control: *E. coli* Rosetta (*DE3*), without the plasmid harbouring FDC, *wt*=*wild-type* FDC. (The image was obtained using ChemiDoc™ Imaging System, selecting the UV filter and the Gel Green application for nucleic acids; colony numbering was introduced using GIMP photo editing software)

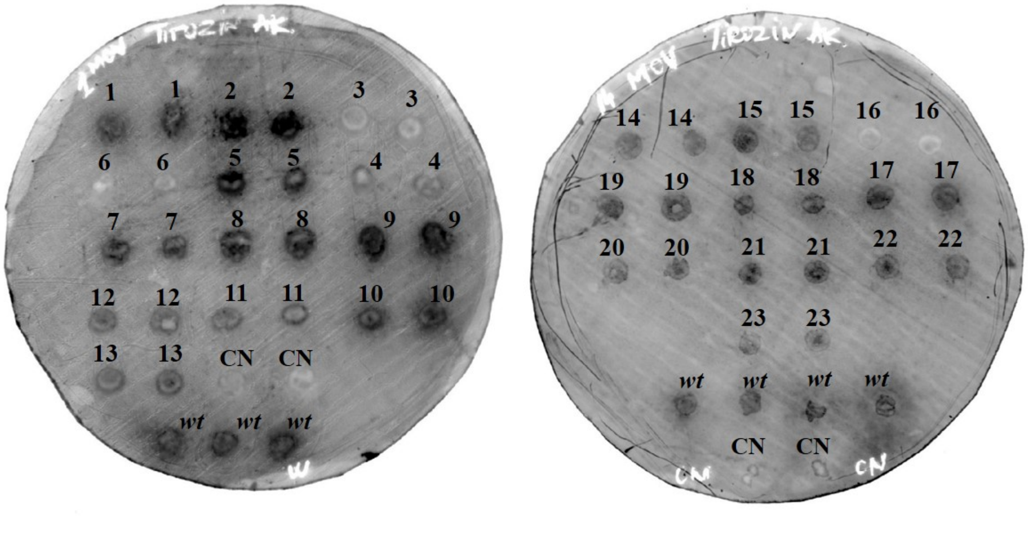

Figure S5. The plate assay showing the fluorescent signals provided by the rationally designed 23 mutant-library of *Sc*FDC (1 to 23) in case of substrate **1d.** CN= negative control: *E. coli* Rosetta (*DE3*), without the plasmid harbouring FDC, *wt*=*wild-type* FDC. (The image was obtained using ChemiDoc™ Imaging System, selecting the UV filter and the Gel Green application for nucleic acids; colony numbering was introduced using GIMP photo editing software)

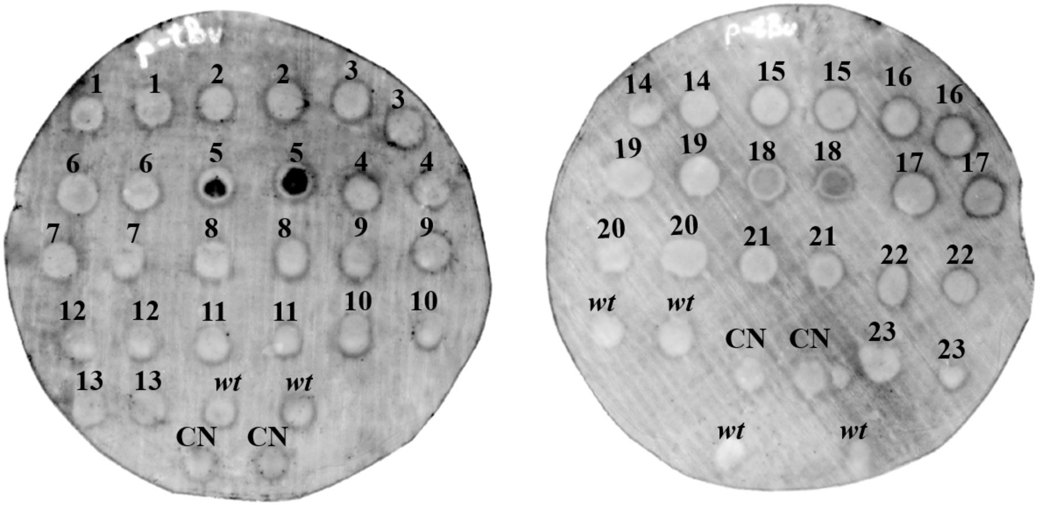

Figure S6. The plate assay showing the fluorescent signals provided by the rationally designed 23 mutant-library of *Sc*FDC (1 to 23) in case of substrate **1e.** CN= negative control: *E. coli* Rosetta (*DE3*), without the plasmid harbouring FDC, *wt*=*wild-type* FDC. (The image was obtained using ChemiDoc™ Imaging System, selecting the UV filter and the Gel Green application for nucleic acids; colony numbering was introduced using GIMP photo editing software)

Table S4. Relative fluorescent signal intensities (%) resulted from the plate-assay of the FDC variant library within the decarboxylation of **1f-j**.

Entry	*Sc*FDC	Substrate 1f-j				
		**1f**	**1g**	**1h**	**1i**	**1j**
1	wt	60	32	34	0	66
2	**M1**-I398A	9	100	97	26	81
3	**M2**-F397A	1	42	0	1	90
4	**M3**-M286A	2	4	0	1	10
5	**M4**-M286V	3	39	53	0	100
6	**M5-**F397V/I398A	9	70	99	28	61
7	**M6**-L442V/F440A	3	5	0	1	8
8	**M7**-I330V/I398A	4	78	98	26	72
9	**M8**-Q192A	2	43	75	0	50
10	**M9**-Q192N	2	51	11	0	59
11	**M10**-Q192S	9	30	30	0	40
12	**M11**-I330A	12	33	100	16	89
13	**M12**-I330V	15	61	36	0	29
14	**M13**-I189A	57	92	95	12	57
15	**M14**-I189V	49	75	0	8	88
16	**M15**-F397Y	29	70	9	4	50
17	**M16**-F397V	9	8	0	0	3
18	**M17**-I398V	48	41	31	0	61
19	**M18-**F397V/I398V	83	57	24	3	45
20	**M19**-I330A/I398A	100	11	18	50	70
21	**M20** -I398V/I189A	55	64	68	18	77
22	**M21 -** I398A/I189A	31	91	100	83	55
23	**M22 -** F397Y/I189V	40	96	99	12	62
24	**M23 -** F397Y/I189A	95	48	100	100	61

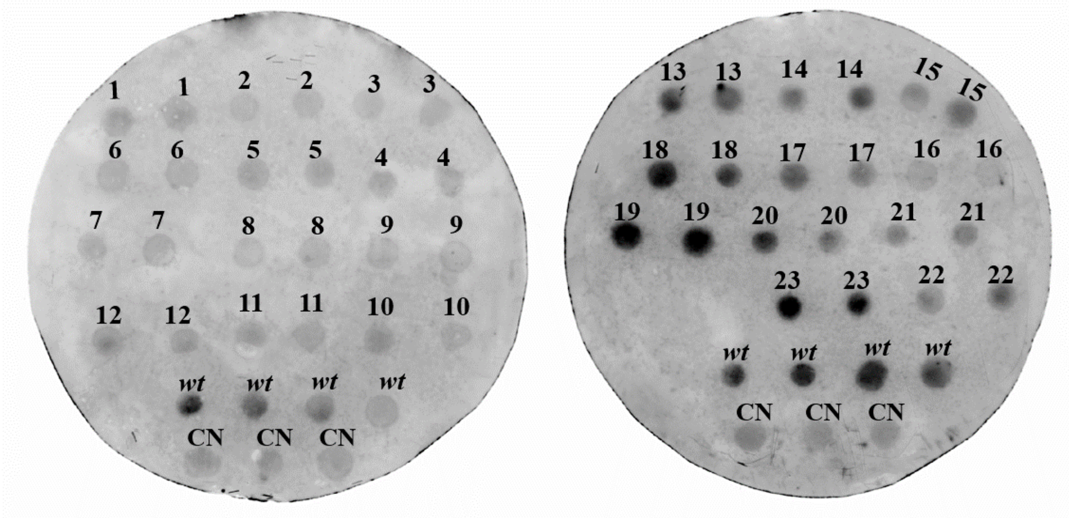

Figure S7. The plate assay showing the fluorescent signals provided by the rationally designed 23 mutant-library of *Sc*FDC (1 to 23) in case of substrate **1f.** CN= negative control: *E. coli* Rosetta (DE3), without the plasmid harbouring FDC, *wt*=*wild-type* FDC. (The image was obtained using ChemiDoc™ Imaging System, selecting the UV filter and the Gel Green application for nucleic acids; colony numbering was introduced using GIMP photo editing software)

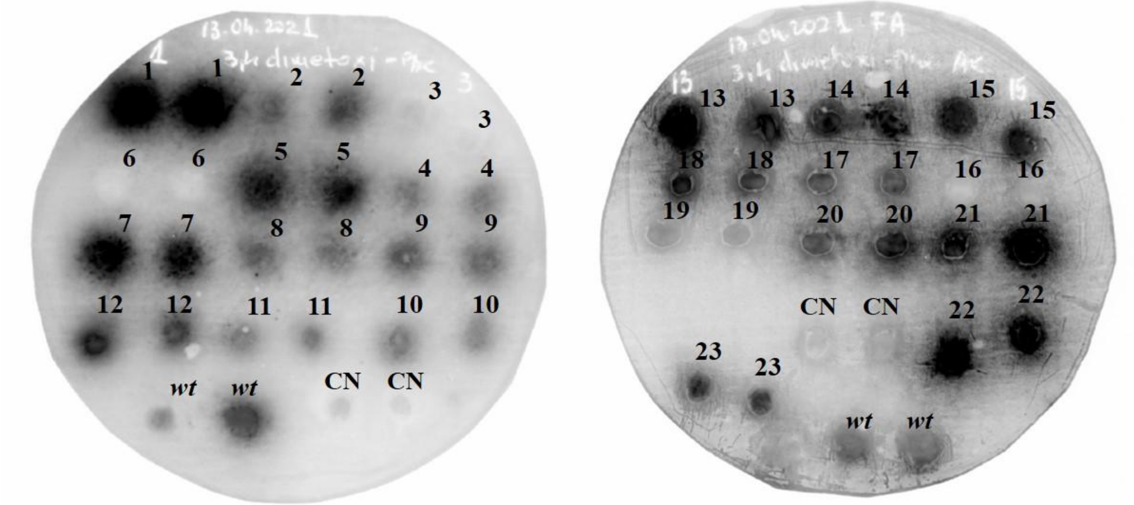

Figure S8. The plate assay showing the fluorescent signals provided by the rationally designed 23 mutant-library of *Sc*FDC (1 to 23) in case of substrate **1g.** CN= negative control: *E. coli* Rosetta (DE3), without the plasmid harbouring FDC, *wt*=*wild-type* FDC. (The image was obtained using ChemiDoc™ Imaging System, selecting the UV filter and the Gel Green application for nucleic acids; colony numbering was introduced using GIMP photo editing software)

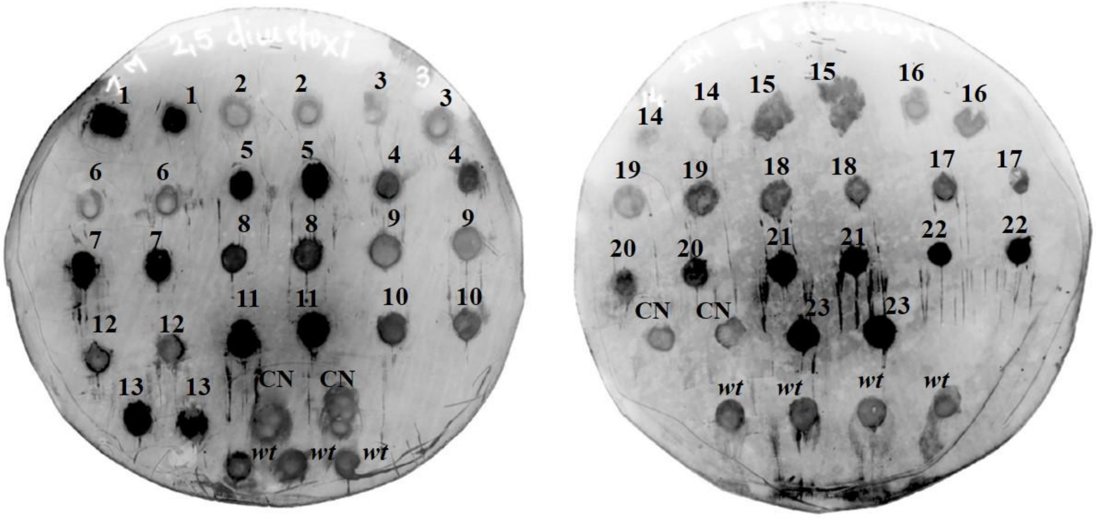

Figure S9. The plate assay showing the fluorescent signals provided by the rationally designed 23 mutant-library of *Sc*FDC (1 to 23) in case of substrate **1h.** CN= negative control: *E. coli* Rosetta (DE3), without the plasmid harbouring FDC, *wt*=*wild-type* FDC. (The image was obtained using ChemiDoc™ Imaging System, selecting the UV filter and the Gel Green application for nucleic acids; colony numbering was introduced using GIMP photo editing software)

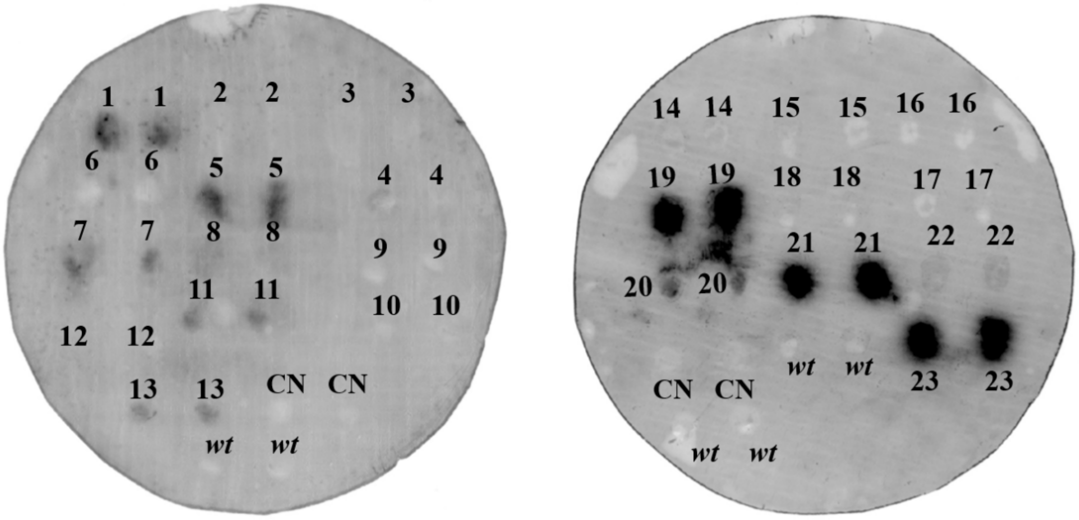

Figure S10. The plate assay showing the fluorescent signals provided by the rationally designed 23 mutant-library of *Sc*FDC (1 to 23) in case of substrate **1i.** CN= negative control: *E. coli* Rosetta (DE3), without the plasmid harbouring FDC, *wt*=*wild-type* FDC. (The image was obtained using ChemiDoc™ Imaging System, selecting the UV filter and the Gel Green application for nucleic acids; colony numbering was introduced using GIMP photo editing software)

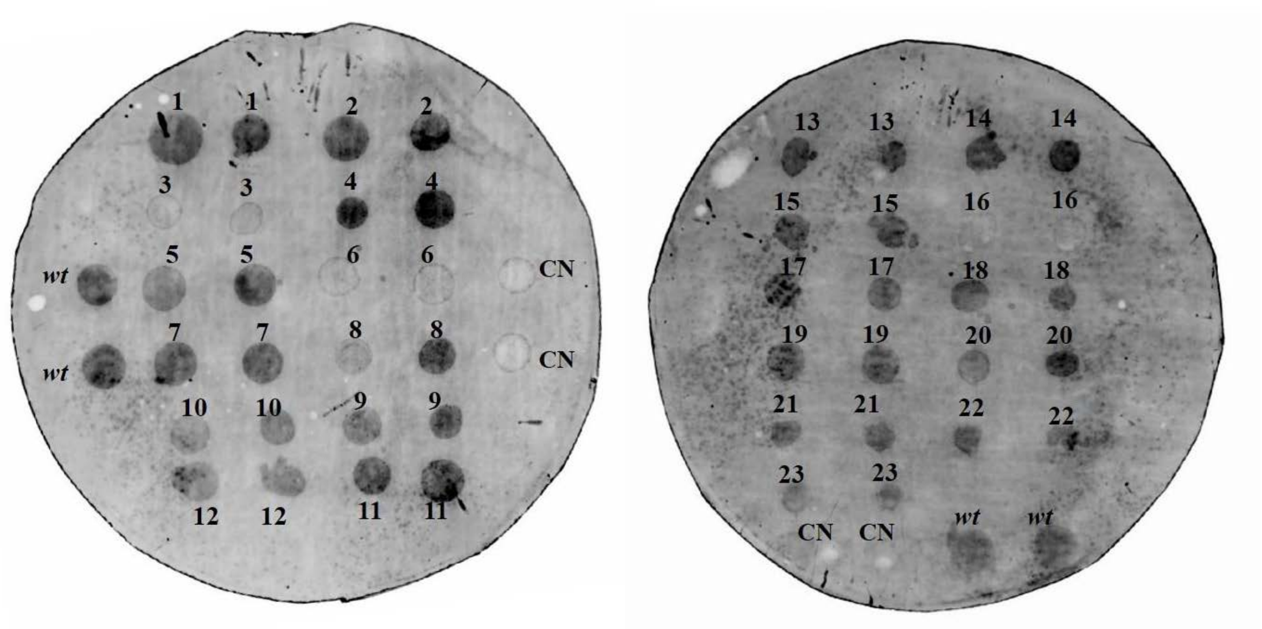

Figure S11. The plate assay showing the fluorescent signals provided by the rationally designed 23 mutant-library of *Sc*FDC (1 to 23) in case of substrate **1j.** CN= negative control: *E. coli* Rosetta (DE3), without the plasmid harbouring FDC, *wt*=*wild-type* FDC. (The image was obtained using ChemiDoc™ Imaging System, selecting the UV filter and the Gel Green application for nucleic acids; colony numbering was introduced using GIMP photo editing software)

Table S5. Relative fluorescent signal intensities (%) resulted from the plate-assay of the FDC variant library within the decarboxylation of **1k-o**.

**Entry**	***Sc*FDC**	**Substrate 1k-o**				
		**1k**	**1l**	**1m**	**1n**	**1o**
1	**wt-FDC**	78	51	59	11	10
2	**M1-**I398A	21	8	53	56	11
3	**M2-**F397A	31	28	61	48	43
4	**M3**-M286A	18	31	0	19	10
5	**M4**-M286V	37	2	11	20	11
6	**M5-**F397V/I398A	42	9	37	65	61
7	**M6**-L442V/F440A	9	70	1	43	27
8	**M7-**I330V/I398A	27	64	29	60	8
9	**M8**-Q192A	61	69	19	75	73
10	**M9**-Q192N	66	61	26	100	12
11	**M10**-Q192S	79	5	24	45	100
12	**M11**-I330A	40	8	21	47	5
13	**M12**-I330V	62	33	28	63	6
14	**M13**-I189A	100	28	53	37	11
15	**M14**-I189V	21	68	75	19	10
16	**M15**-F397Y	18	97	31	71	53
17	**M16**-F397V	5	89	5	9	12
18	**M17**-I398V	5	100	21	23	39
19	**M18**-F397V/I398V	9	59	100	60	93
20	**M19**-I330A/I398A	8	62	59	21	8
21	**M20** -I398V/I189A	34	61	35	51	9
22	**M21** - I398A/I189A	31	11	37	82	9
23	**M22** - F397Y/I189V	30	13	37	59	12
24	**M23** - F397Y/I189A	45	60	27	15	2

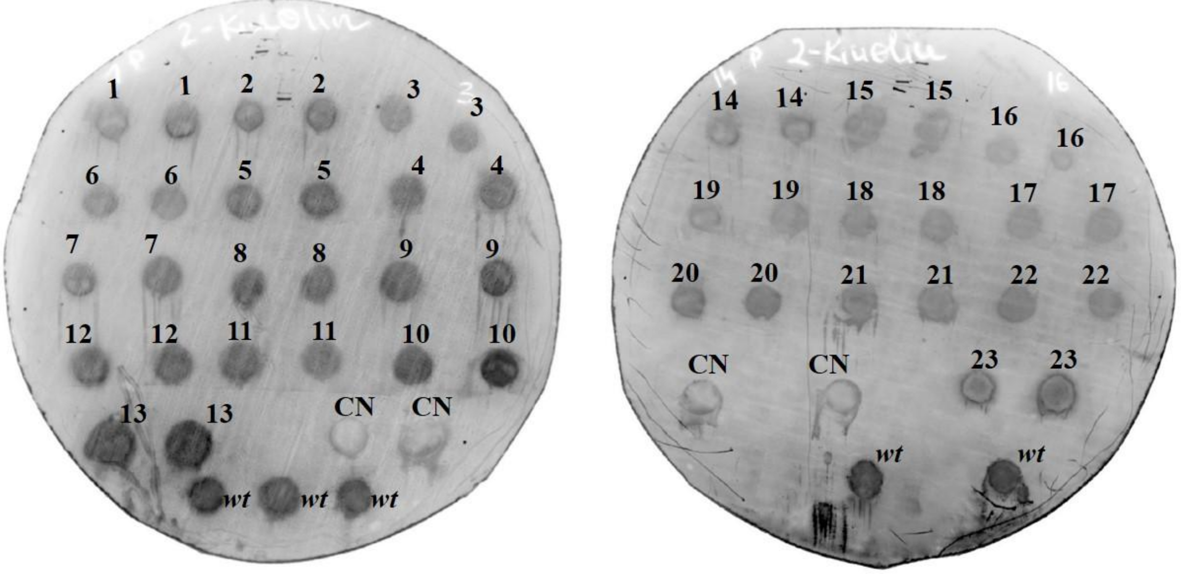

Figure S12. The plate assay showing the fluorescent signals provided by the rationally designed 23 mutant-library of *Sc*FDC (1 to 23) in case of substrate **1k.** CN= negative control: *E. coli* Rosetta (DE3), without the plasmid harbouring FDC, *wt*=*wild-type* FDC. (The image was obtained using ChemiDoc™ Imaging System, selecting the UV filter and the Gel Green application for nucleic acids; colony numbering was introduced using GIMP photo editing software)

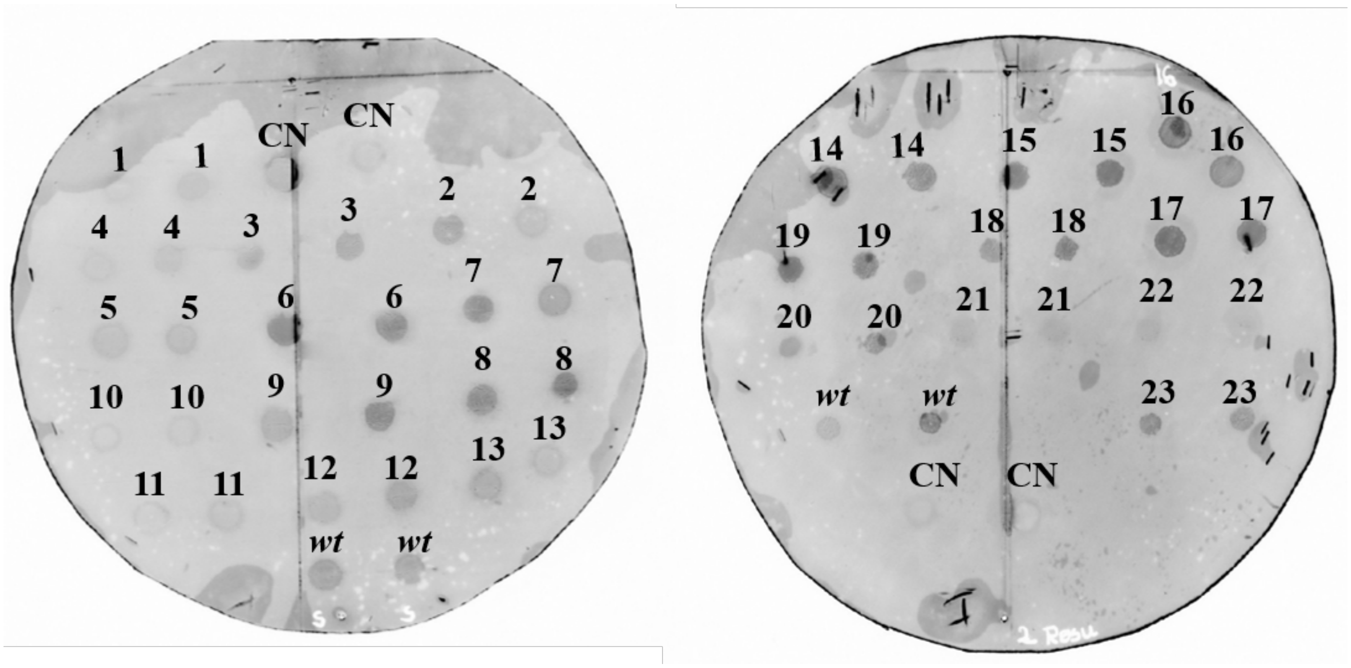

Figure S13. The plate assay showing the fluorescent signals provided by the rationally designed 23 mutant-library of *Sc*FDC (1 to 23) in case of substrate **1l.** CN= negative control: *E. coli* Rosetta (DE3), without the plasmid harbouring FDC, *wt*=*wild-type* FDC. (The image was obtained using ChemiDoc™ Imaging System, selecting the UV filter and the Gel Green application for nucleic acids; colony numbering was introduced using GIMP photo editing software)

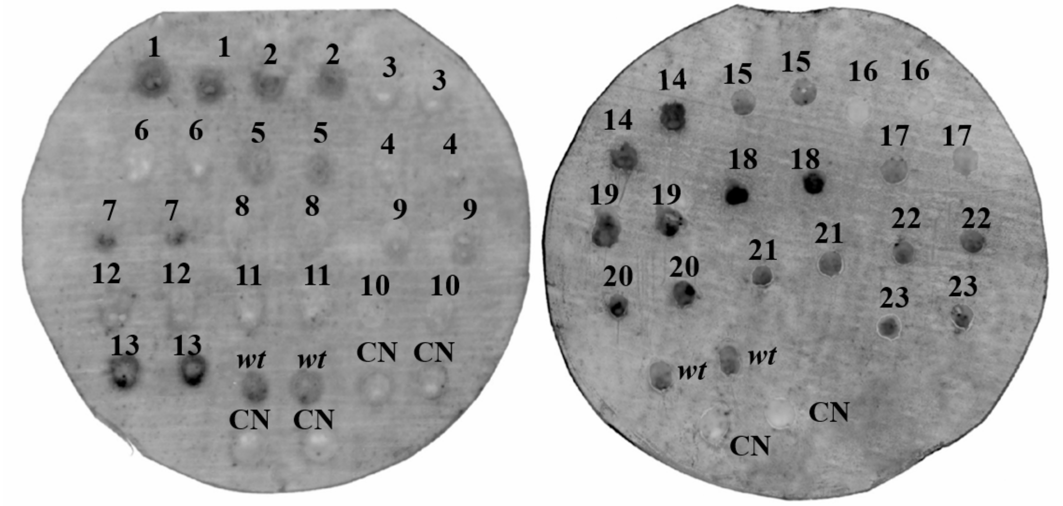

Figure S14. The plate assay showing the fluorescent signals provided by the rationally designed 23 mutant-library of *Sc*FDC (1 to 23) in case of substrate **1m.** CN= negative control: *E. coli* Rosetta (*DE3*), without the plasmid harbouring FDC, *wt*=*wild-type* FDC. (The image was obtained using ChemiDoc™ Imaging System, selecting the UV filter and the Gel Green application for nucleic acids; colony numbering was introduced using GIMP photo editing software)

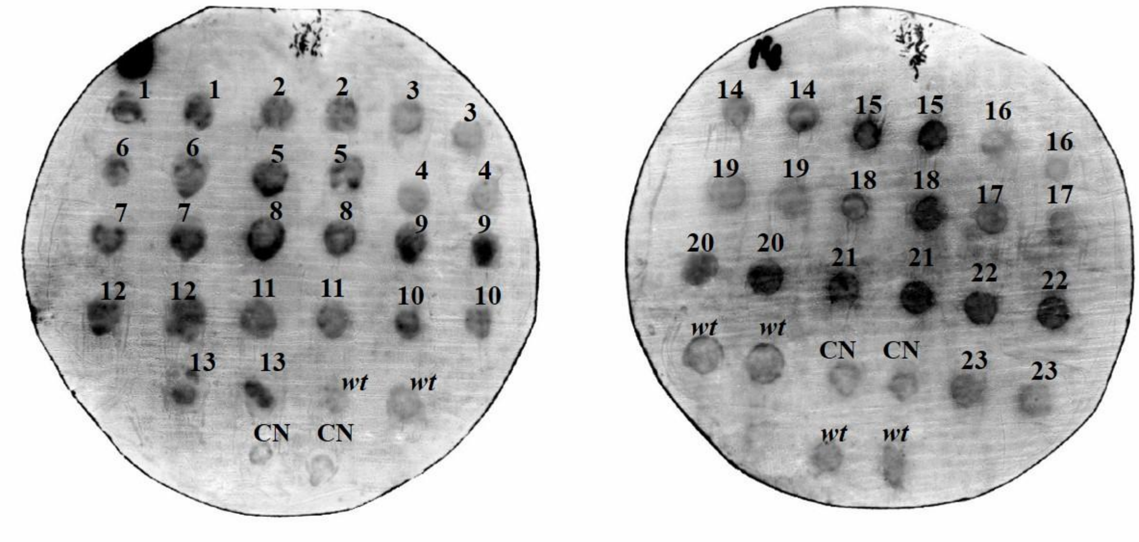

Figure S15. The plate assay showing the fluorescent signals provided by the rationally designed 23 mutant-library of *Sc*FDC (1 to 23) in case of substrate **1n.** CN= negative control: *E. coli* Rosetta (DE3), without the plasmid harbouring FDC, *wt*=*wild-type* FDC. (The image was obtained using ChemiDoc™ Imaging System, selecting the UV filter and the Gel Green application for nucleic acids; colony numbering was introduced using GIMP photo editing software)

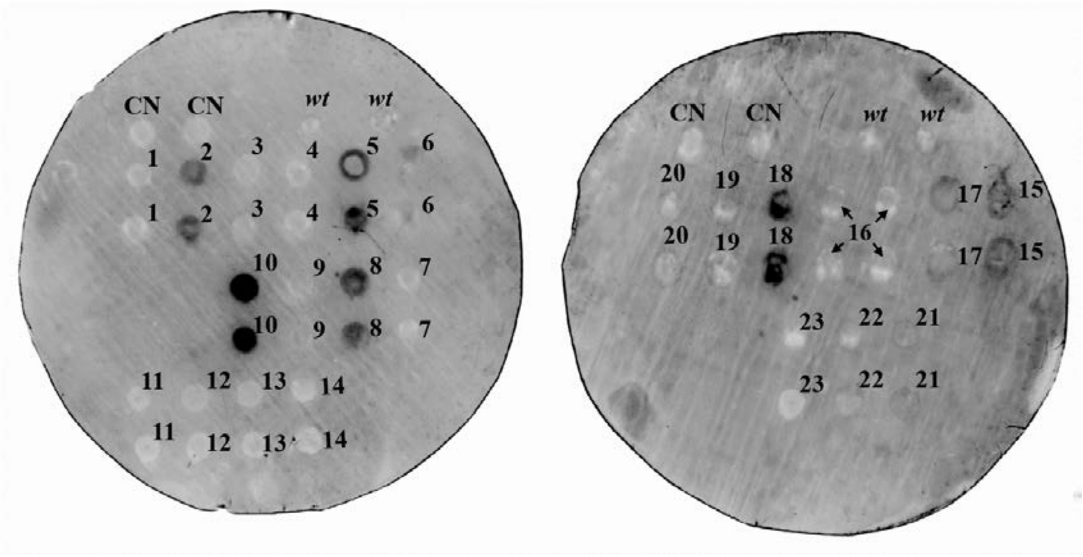

Figure S16. The plate assay showing the fluorescent signals provided by the rationally designed 23 mutant-library of *Sc*FDC (1 to 23) in case of substrate **1o.** CN= negative control: *E. coli* Rosetta (*DE3*), without the plasmid harbouring FDC, *wt*=*wild-type* FDC. (The image was obtained using ChemiDoc™ Imaging System, selecting the UV filter and the Gel Green application for nucleic acids; colony numbering was introduced using GIMP photo editing software)

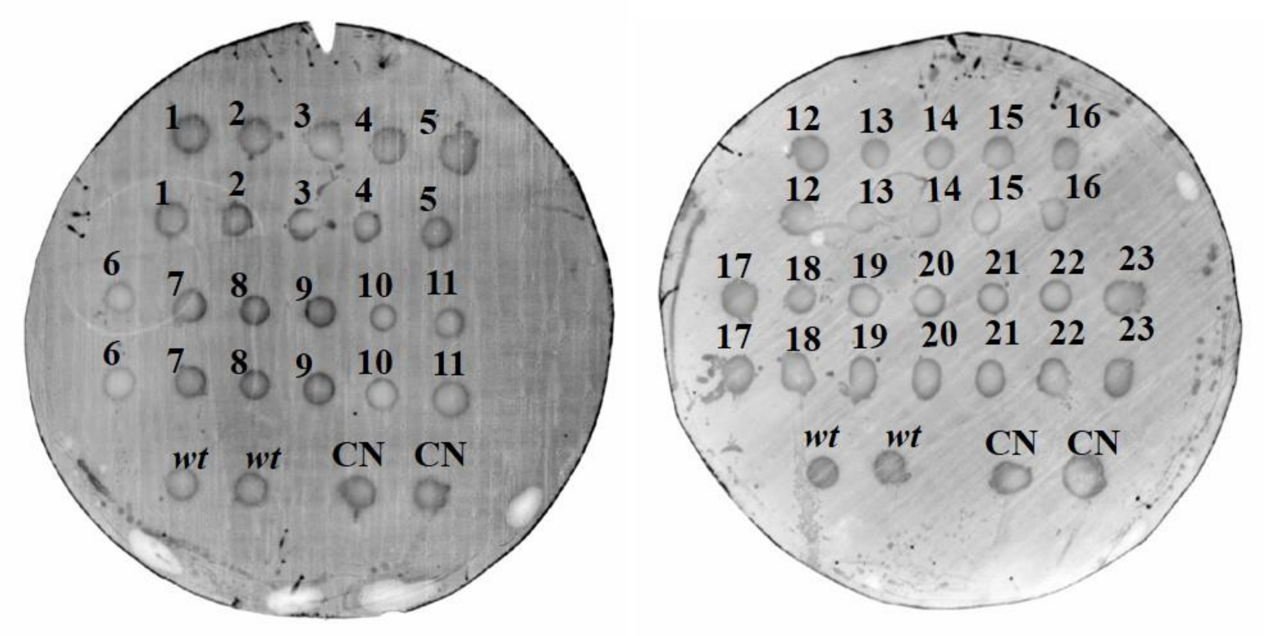

Figure S17. The plate assay showing the fluorescent signals provided by the rationally designed 23 mutant-library of *Sc*FDC (1 to 23) in case of substrate **1p.** CN= negative control: *E. coli* Rosetta (DE3), without the plasmid harbouring FDC, *wt*=*wild-type* FDC. (The image was obtained using ChemiDoc™ Imaging System, selecting the UV filter and the Gel Green application for nucleic acids; colony numbering was introduced using GIMP photo editing software).

7. HPLC methods

HPLC samples were prepared by diluting 100 μL of reaction mixture with 100 μL of a solution containing: *a)* 50% v/v 100 mM NaH_2PO_4 buffer, pH 7.0, 42.5% v/v MeCN and 7.5% v/v of 2.5 mg/ml benzalacetophenone (internal standard) solution in MeCN, in case of substrates **1a-g**, **1i-j** and **1o**, *b)* 50% v/v 100 mM NaH_2PO_4 buffer, pH 7.0, 47.5% v/v MeCN and 2.5% v/v of 2.5 mg/ml benzalacetophenone (internal standard) solution in MeCN for substrates **1h** and **1n**, *c)* 50% v/v 100 mM NaH_2PO_4 buffer, pH 7.0, 35% v/v MeCN and 15% v/v of 2.5 mg/ml benzalacetophenone (internal standard) solution in MeCN for substrate **1k**, *d)* 50% v/v 100 mM mM NaH_2PO_4 buffer, pH 7.0, 30% v/v MeCN and 20% v/v of 2.5 mg/ml benzalacetophenone (internal standard) solution in MeCN for substrate **1l**, *e)* 50% v/v NaH_2PO_4 buffer, pH 7.0, 40% v/v MeCN and 10% v/v of 2.5 mg/ml benzalacetophenone (internal standard) solution in MeCN for substrate **1m**. HPLC analysis were performed on Agilent 1200 or 1260 HPLC systems using Phenomenex NX-C18 150x4.5 mm columns, injecting 5 μL of the solutions obtained as described above. The mobile phase was a mixture of 70% MeCN (0.1% v/v TFA) and 30% H_2O (0.1% v/v TFA) and flow rate of 1 mL/min. Conversion values were determined based on the depletion of the substrate concentration, measured using benzalacetophenone as an internal standard.

Table S6. HPLC retention times and response factors

**Substrate**	**Retention time (min)**		**Relative response factors 1a-p *vs* int. stand.**	**Wavelength of UV detection (nm)**
	**Substrates**	**Internal standard**		
(*E*)-3-(2-ethoxyphenyl)acrylic acid **- 1a**	2.2	3.7	1.69	270
(*E*)-3-(3-ethoxyphenyl)acrylic acid **- 1b**	2.1	3.7	2.00	270
(*E*)-3-(4-ethoxyphenyl)acrylic acid **- 1c**	2.1	3.6	1.26	310
(*E*)-3-(4-hydroxyphenyl)acrylic acid - **1d**	1.7	3.4	0.89	310
(*E*)-3-(4-(*tert*-butyl)phenyl)acrylic acid - **1e**	2.7	3.7	1.57	290
(*E*)-3-(3-bromo-4-methoxyphenyl)acrylic acid - **1f**	2.1	3.7	0.98	290
(*E*)-3-(3,4-dimethoxyphenyl)acrylic acid - **1g**	1.8	3.3	1.25	330
(*E*)-3-(2,5-dimethoxyphenyl)acrylic acid - **1h**	2.0	3.5	0.23	330
(*E*)-3-(3,4,5-trimethoxyphenyl)acrylic acid - **1i**	1.9	3.8	0.76	300
(*E*)-3-(benzofuran-3-yl)acrylic acid - **1j**	2.1	3.6	1.82	270
(*E*)-3-(quinolin-2-yl)acrylic acid - **1k**	1.5	3.6	3.54	260
(*E*)-3-([1,1'-biphenyl]-2-yl)acrylic acid - **1l**	2.6	3.7	2.95	260
(*E*)-3-([1,1'-biphenyl]-3-yl)acrylic acid - **1m**	2.6	3.7	4.83	260
(*E*)-3-(4'-bromo-[1,1'-biphenyl]-4-yl)acrylic acid - **1n**	3.3	3.6	0.40	310
(*E*)-3-(4-phenoxyphenyl)acrylic acid - **1o**	2.6	3.7	1.14	290
(*E*)-3-(10-methyl-10*H*-phenothiazin-3-yl)acrylic acid - **1p**	2.8	3.5	n. d.	250

Eluent: 70% MeCN (0.1% v/v TFA), 30% H_2O (0.1% v/v TFA); flow rate: 1 mL/min, 25°C, column: Phenomenex NX-C18 150x4.5 mm, injection volume: 5μL

Table S7. Conversions of the decarboxylation of (*E*)-3-(2-ethoxyphenyl)acrylic acid (**1a**) using the FDC variants selected from the plate-assay.

Entry	Conversion (%)	Relative fluorescence intensity (%)– plate assay
Wild-type	62.1	35
M14 - I189V	97.8	98
M13 - I189A	94.4	100
M17 - I398V	70.7	37
M1 - I398A	42.8	28
M19 - I330A/I398A	37.8	9

Table S8. Conversions of the decarboxylation of (*E*)-3-(3-ethoxyphenyl)acrylic acid (**1b**) using the FDC variants selected from the plate-assay.

Entry	Conversion (%)	Relative fluorescence intensity (%)– plate assay
Wild-type	70.6	61
M13 - I189A	>99.9	100
M14 - I189V	>99.9	66
M20 - I398V/I189A	>99.9	53
M2 - F397A	56.3	84
M5 - F397V/I398A	36.5	51
M7 - I330V/I398A	25.2	34
M9 - Q192N	16.2	31
M16 - F397V	11.5	1
M3 - M286A	8.0	0

Table S9. Conversions of the decarboxylation of (*E*)-3-(4-ethoxyphenyl)acrylic acid (**1c**) using the FDC variants selected from the plate-assay.

Entry	Conversion (%)	Relative fluorescence intensity (%)– plate assay
Wild-type	39.5	47
M1 - I398A	92.7	88
M5 - F397V/I398A	91.4	77
M9 - Q192N	78.3	100
M2 - F397A	77.7	90
M15 - F397Y	69.4	28
M18 - F397V/I398V	69.1	58
M14 - I189V	47.3	25
M16 - F397V	<1	0

Table S10. Conversions of the decarboxylation of (*E*)-3-(4-hydroxyphenyl)acrylic acid (**1d**) using the FDC variants selected from the plate-assay.

**Entry**	**Conversion (%)**	**Relative fluorescence intensity (%)– plate assay**
Wild-type	22.4	33
M2 - F397A	50.9	100
M9 - Q192N	44.7	80
M8 - Q192A	38.9	65
M17 - I398V	23.8	47
M7 - I330V/I398A	20.9	51
M14 - I189V	8.7	21
M16 - F397V	<1	0

Table S11. Conversions of the decarboxylation of (*E*)-3-(4-(*tert*-butyl)phenyl)acrylic acid (**1e**) using the FDC variants selected from the plate-assay.

**Entry**	**Conversion (%)**	**Relative fluorescence intensity (%)– plate assay**
Wild-type	<1	0
M5 - F397V/I398A	24.2	100
M18 - F397V/I398V	11.3	43

Table S12. Conversions of the decarboxylation of (*E*)-3-(3-bromo-4-methoxyphenyl)acrylic acid (**1f**) using the FDC variants selected from the plate-assay.

**Entry**	**Conversion (%)**	**Relative fluorescence intensity (%)– plate assay**
Wild-type	66.0	60
M19 - I330A/I398A	83.9	100
M14 - I189V	75.9	49
M13 - I189A	73.2	57
M17 - I398V	72.5	48
M20 - I398V/I189A	65.0	55
M15 - F397Y	64.5	29
M23 - F397Y/I189A	44.4	95
M7 - I330V/I398A	41.5	4
M16 - F397V	32.8	9
M11 - I330A	15.9	12
M1 - I398A	12.2	9

Table S13. Conversions of the decarboxylation of (*E*)-3-(3,4-dimethoxyphenyl)acrylic acid (**1g**) using the FDC variants selected from the plate-assay.

**Entry**	**Conversion (%)**	**Relative fluorescence intensity (%)– plate assay**
Wild-type	14.4	32
M20 - I398V/I189A	67.0	64
M23 - F397Y/I189A	65.4	48
M21 - I398A/I189A	60.3	91
M5 - F397V/I398A	60.3	70
M13 - I189A	60.0	92
M14 - I189V	59.4	75
M1 - I398A	58.5	100
M22 - F397Y/I189V	44.4	96
M7 - I330V/I398A	43.5	78
M15 - F397Y	42.6	70
M10 - Q192S	11.2	30
M11 - I330A	10.0	33
M19 - I330A/I398A	3.4	11
M6 - L442V/F440A	<1	5

Table S14. Conversions of the decarboxylation of (*E*)-3-(2,5-dimethoxyphenyl)acrylic acid (**1h**) using the FDC variants selected from the plate-assay.

**Entry**	**Conversion (%)**	**Relative fluorescence intensity (%)– plate assay**
Wild-type	33.1	34
M23 - F397Y/I189A	78.8	100
M22 - F397Y/I189V	75.8	99
M11 - I330A	72.7	100
M21 - I398A/I189A	65.9	100
M13 - I189A	61.7	95
M20 - I398V/I189A	53.8	68
M1 - I398A	50.2	97
M5 - F397V/I398A	48.9	99
M7 - I330V/I398A	45.5	98
M8 - Q192A	38.4	75
M19 - I330A/I398A	31.8	18
M2 - F397A	<1	0
M3 - M286A	<1	0
M6 - L442V/F440A	<1	0

Table S15. Conversions of the decarboxylation of (*E*)-3-(3,4,5-trimethoxyphenyl)acrylic acid (**1i**) using the FDC variants selected from the plate-assay.

**Entry**	**Conversion (%)**	**Relative fluorescence intensity (%)– plate assay**
Wild-type	<1	0
M23 - F397Y/I189A	82.7	100
M21 - I398A/I189A	77.5	83
M11 - I330A	66.0	16
M19 - I330A/I398A	65.2	50
M5 - F397V/I398A	59.9	28
M1 - I398A	57.4	26
M7 - I330V/I398A	56.9	26
M20 - I398V/I189A	54.2	18
M6 - L442V/F440A	<1	1
M10 - Q192S	<1	0

Table S16. Conversions of the decarboxylation of (*E*)-3-(benzofuran-3-yl)acrylic acid (**1j**) using the FDC variants selected from the plate-assay.

**Entry**	**Conversion (%)**	**Relative fluorescence intensity (%)– plate assay**
Wild-type	28.6	66
M14 - I189V	89.7	88
M11 - I330A	89.6	89
M17 - I398V	89.2	61
M13 - I189A	84.1	57
M22 - F397Y/I189V	81.1	62
M7 - I330V/I398A	76.5	72
M1 - I398A	74.7	81
M20 - I398V/I189A	71.5	77
M4 - M286V	65.1	100
M9 - Q192N	53.9	59
M2 - F397A	20.5	90
M5 - F397V/I398A	10.3	61
M6 - L442V/F440A	1.4	8
M16 - F397V	<1	3

Table S17. Conversions of the decarboxylation of (*E*)-3-(quinolin-2-yl)acrylic acid (**1k**) using the FDC variants selected from the plate-assay.

Entry	Conversion (%)	Relative fluorescence intensity (%)– plate assay
Wild-type	38.7	78
M13 - I189A	62.9	100
M9 - Q192N	58.6	66
M10 - Q192S	54.1	79
M8 - Q192A	52.7	61
M11 - I330A	45.2	40
M5 - F397V/I398A	30.9	42
M2 - F397A	30.4	31
M15 - F397Y	17.1	18
M17 - I398V	11.7	5
M3 - M286A	6.4	18
M1 - I398A	2.3	21

Table S18. Conversions of the decarboxylation of (*E*)-3-([1,1'-biphenyl]-2-yl)acrylic acid (**1l**) using the FDC variants selected from the plate-assay.

Entry	Conversion (%)	Relative fluorescence intensity (%)– plate assay
Wild-type	11.8	51
M17 - I398V	42.9	100
M15 - F397Y	35.3	97
M9 - Q192N	27.8	61
M16 - F397V	27.6	89
M7 - I330V/I398A	27.4	64
M14 - I189V	24.9	68
M19 - I330A/I398A	21.9	62
M8 - Q192A	19.2	69
M4 - M286V	3.7	2
M10 - Q192S	<1	5

Table S19. Conversions of the decarboxylation of (*E*)-3-([1,1'-biphenyl]-3-yl)acrylic acid (**1m**) using the FDC variants selected from the plate-assay.

**Entry**	**Conversion (%)**	**Relative fluorescence intensity (%)– plate assay**
Wild-type	23.5	59
M2 - F397A	79.0	61
M14 - I189V	73.8	75
M18 - F397V/I398V	65.0	100
M13 - I189A	59.1	53
M8 - Q192A	58.7	19
M16 - F397V	16.7	5
M9 - Q192N	8.7	26
M19 - I330A/I398A	7.2	59
M5 - F397V/I398A	4.5	37

Table S20. Conversions of the decarboxylation of (*E*)-3-(4'-bromo-[1,1'-biphenyl]-4-yl)acrylic acid (**1n**) using the FDC variants selected from the plate-assay.

**Entry**	**Conversion (%)**	**Relative fluorescence intensity (%)– plate assay**
Wild-type	<1	11
M9 - Q192N	51.4	100
M15 - F397Y	41.5	71
M18 - F397V/I398V	37.1	60
M22 - F397Y/I189V	35.5	59
M6 - L442V/F440A	34.0	43
M8 - Q192A	27.2	75
M19 - I330A/I398A	<1	21
M23 - F397Y/I189A	<1	15

Table S21. Conversions of the decarboxylation of (*E*)-3-(4-phenoxyphenyl)acrylic acid (**1o**) using the FDC variants selected from the plate-assay.

Entry	Conversion (%)	Relative fluorescence intensity (%)– plate assay
Wild-type	3.1	10
M8 - Q192A	81.4	73
M15 - F397Y	65.3	53
M5 - F397V/I398A	64.3	61
M10 - Q192S	56.3	100
M2 - F397A	17.4	43
M18 - F397V/I398V	15.3	93
M17 - I398V	12.2	39
M3 - M286A	10.8	10
M11 - I330A	<1	5
M19 - I330A/I398A	<1	8

- **Representative HPLC chromatograms from the separation of 1a-p and benzalacetophenone**

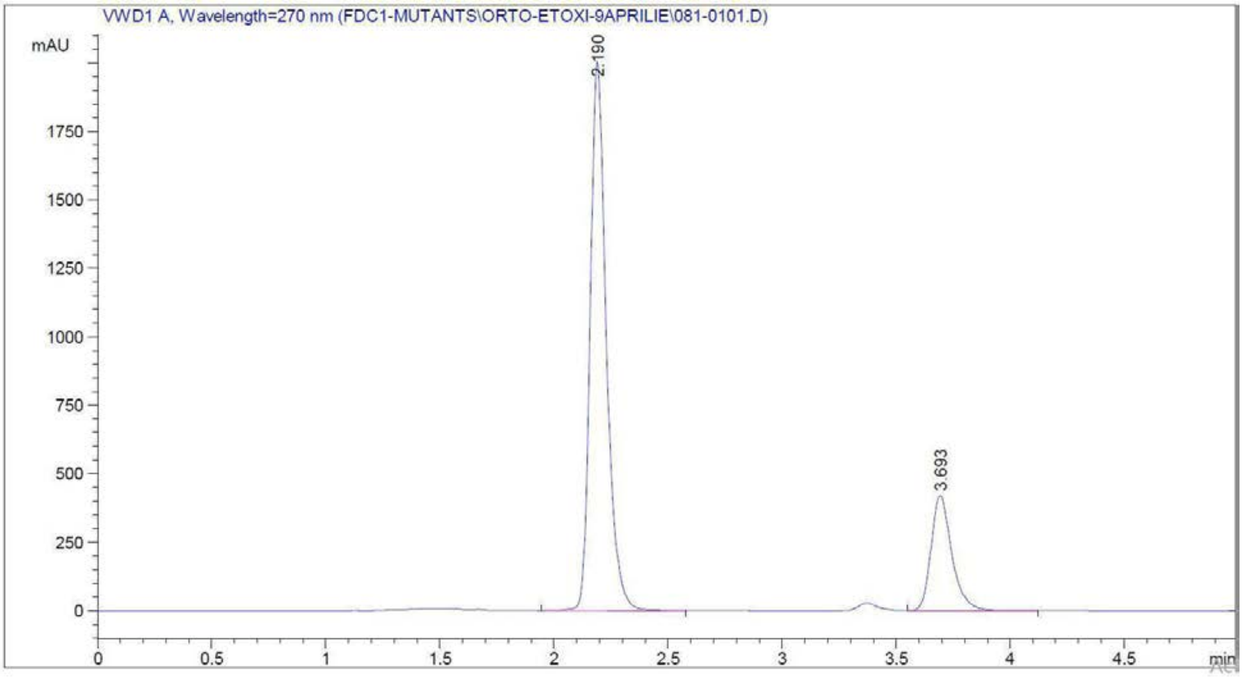

Figure S18. Chromatogram from the HPLC separation of benzalacetophenone and **1a**

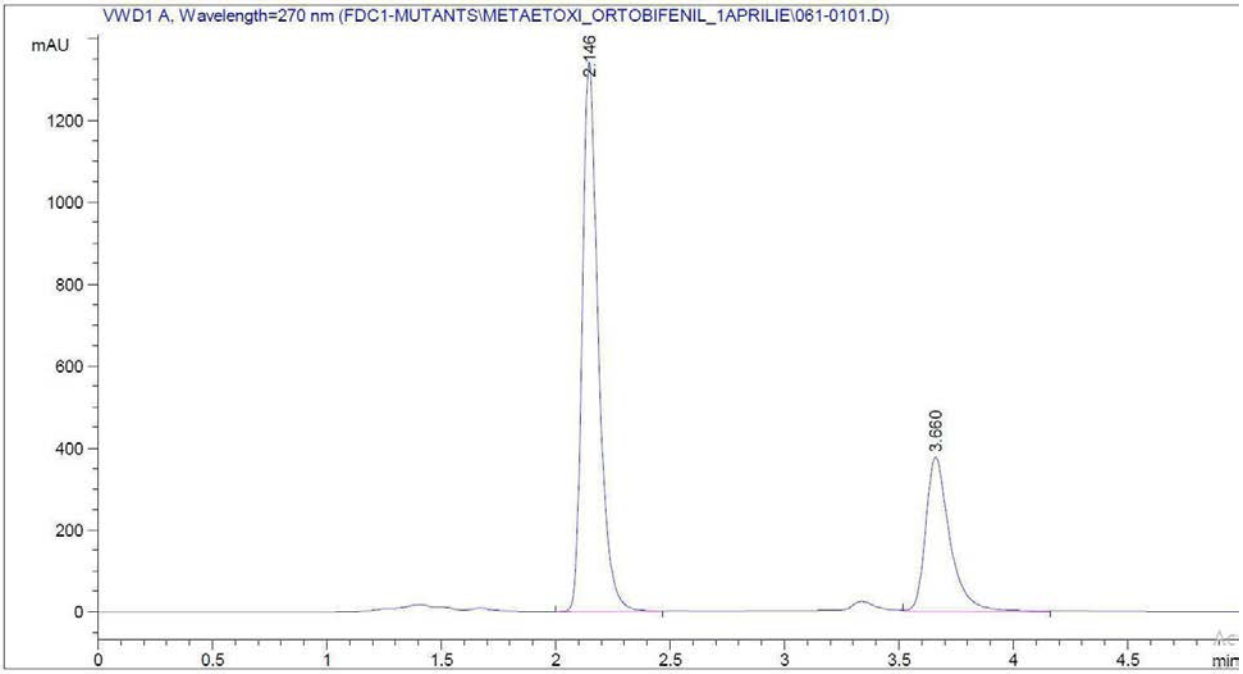

Figure S19. Chromatogram from the HPLC separation of benzalacetophenone and **1b**

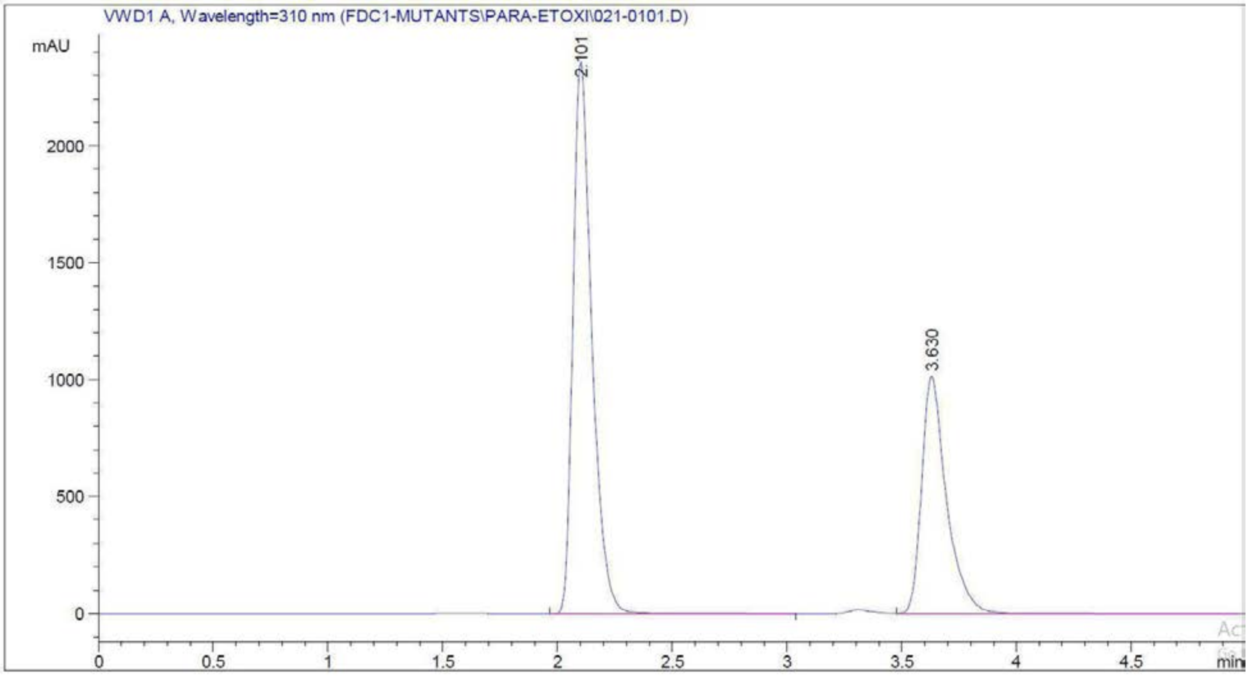

Figure S20. Chromatogram from the HPLC separation of benzalacetophenone and **1c**

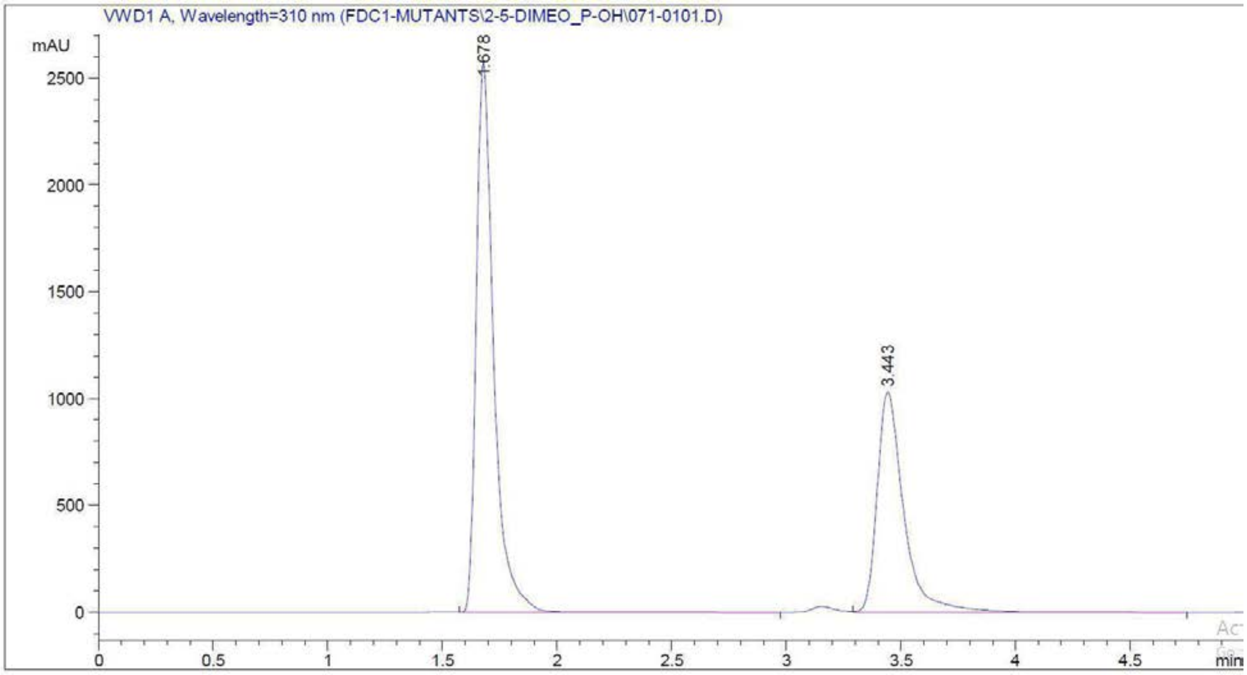

Figure S21. Chromatogram from the HPLC separation of benzalacetophenone and **1d**

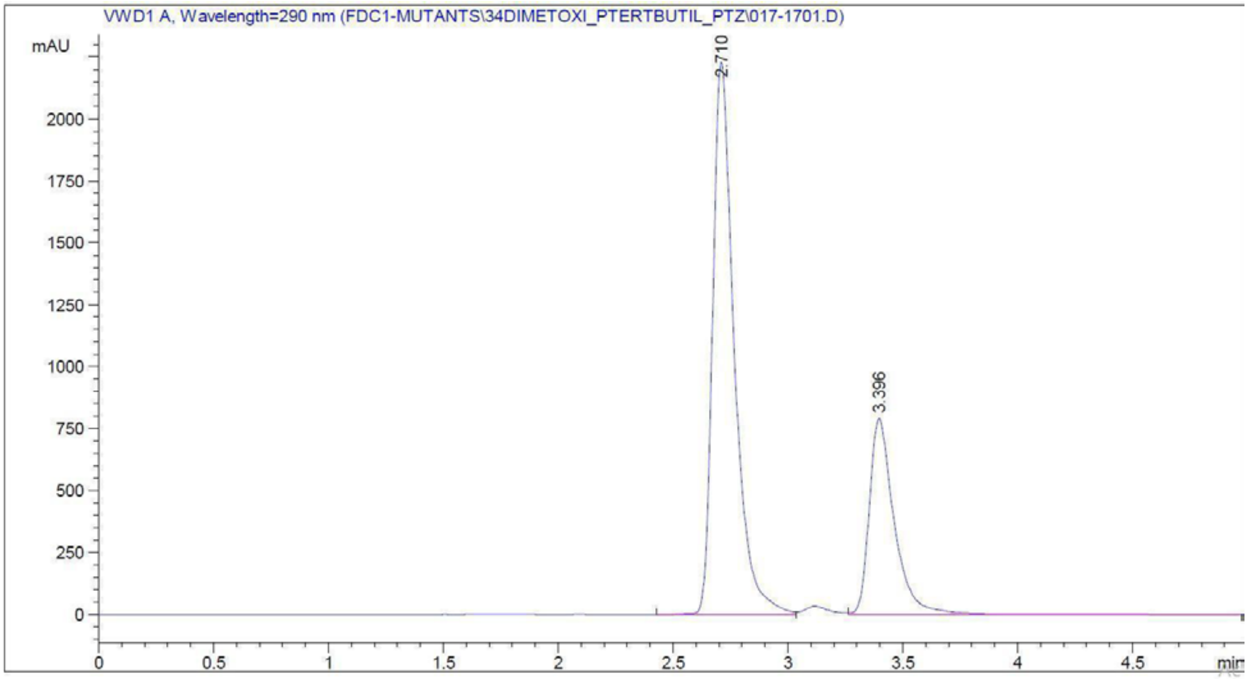

Figure S22. Chromatogram from the HPLC separation of benzalacetophenone and **1e**

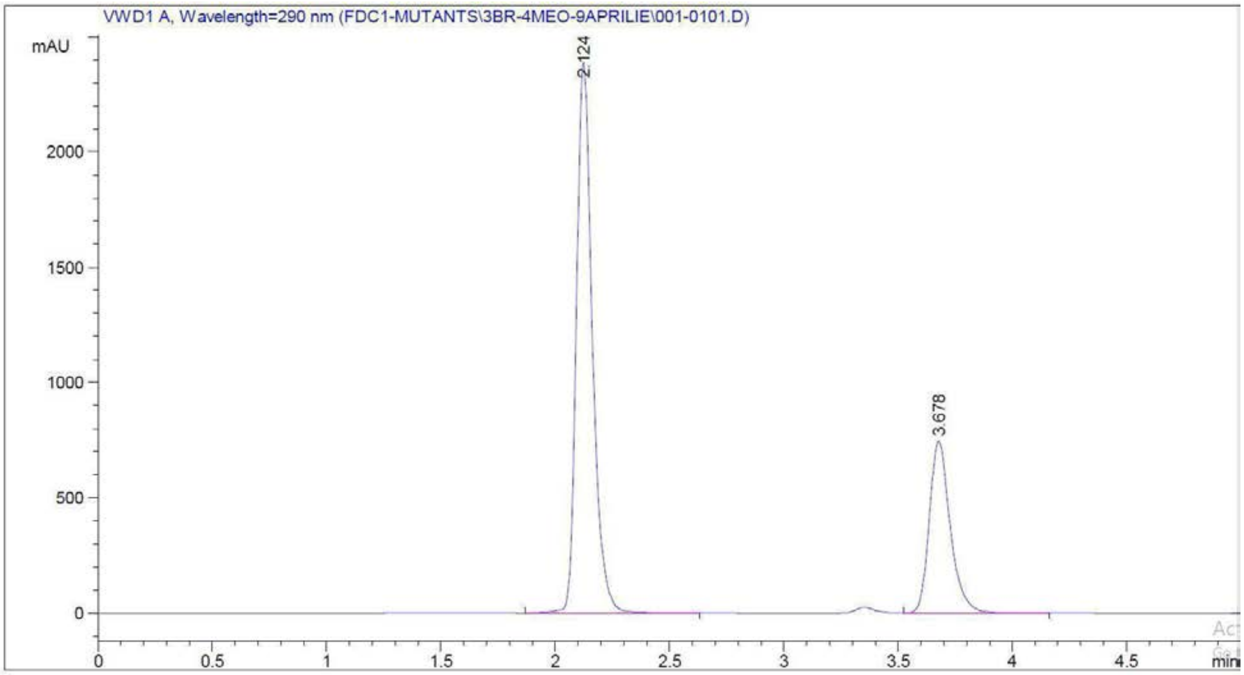

Figure S23. Chromatogram from the HPLC separation of benzalacetophenone and **1f**

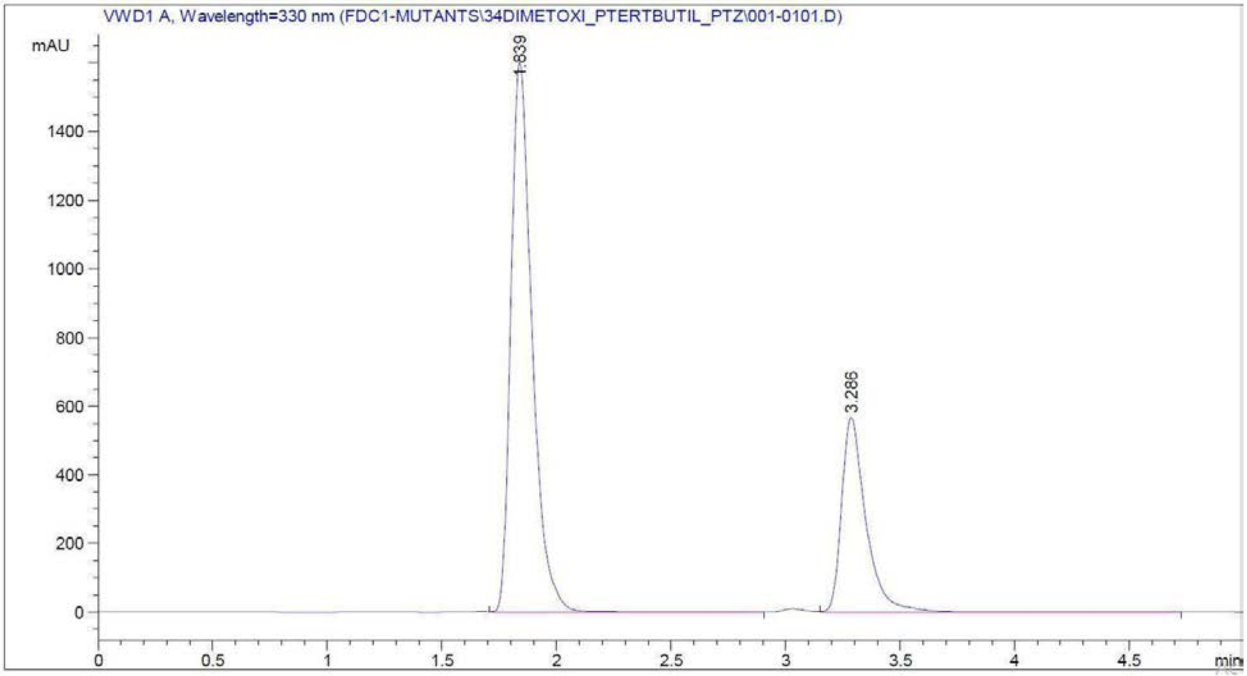

Figure S24. Chromatogram from the HPLC separation of benzalacetophenone and **1g**

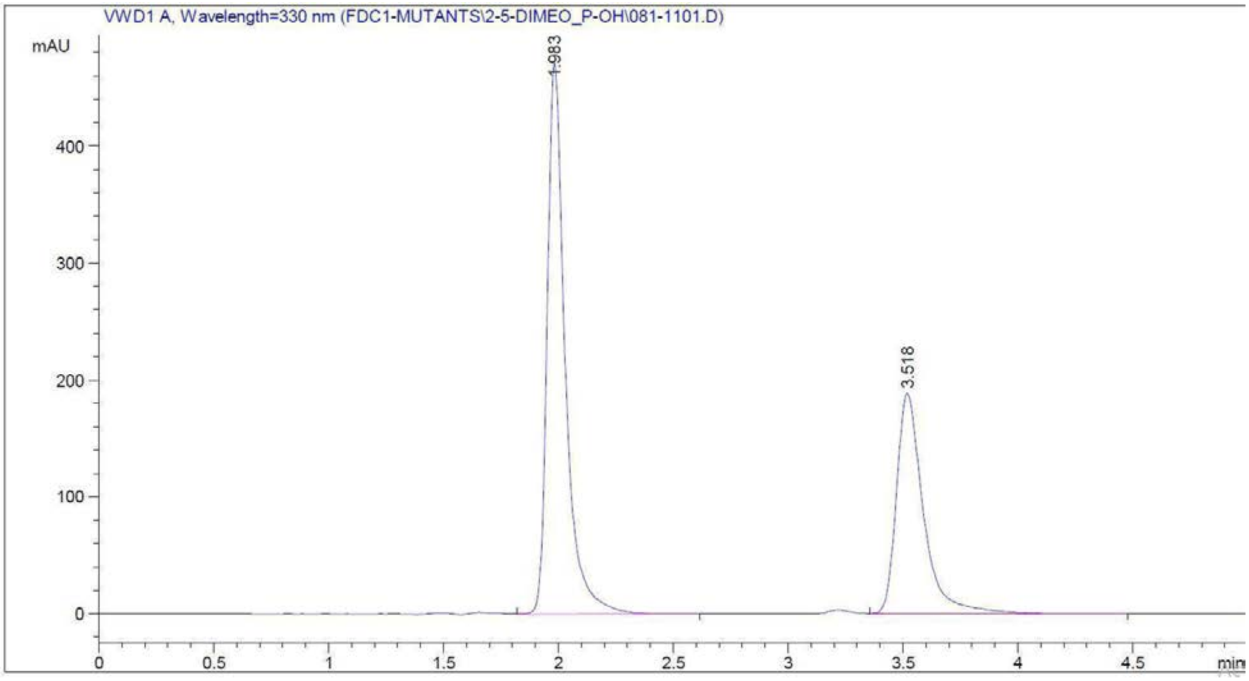

Figure S25. Chromatogram from the HPLC separation of benzalacetophenone and **1h**

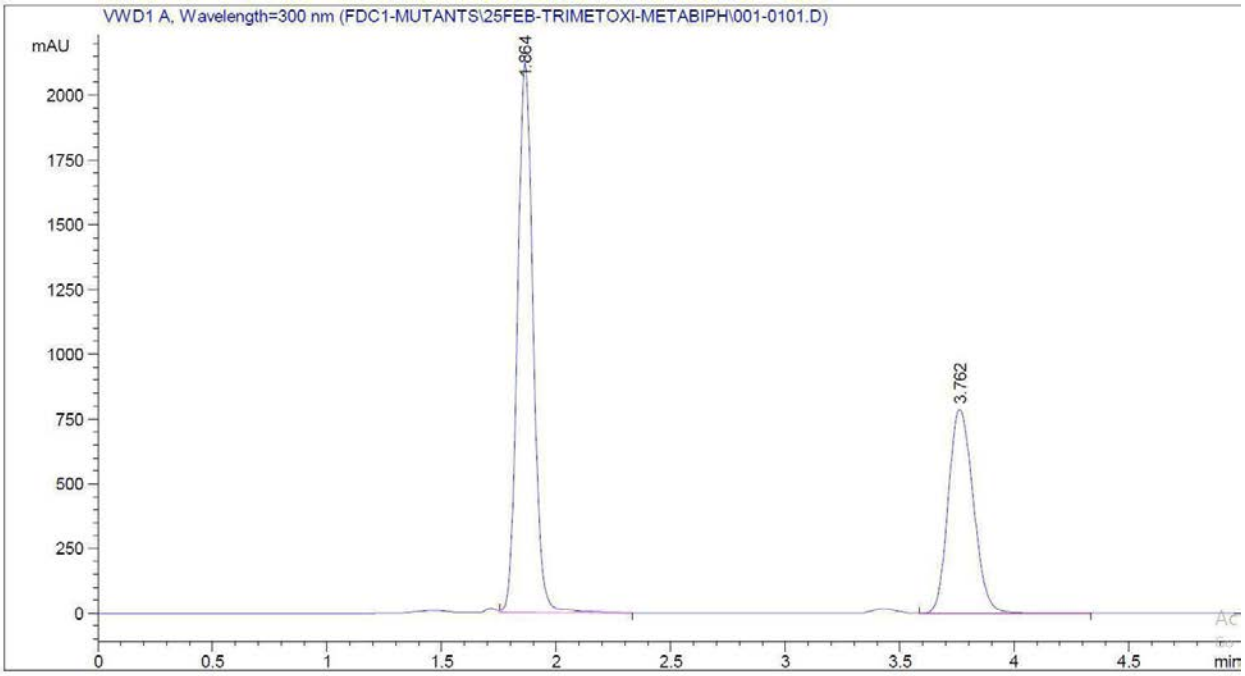

Figure S26. Chromatogram from the HPLC separation of benzalacetophenone and **1i**

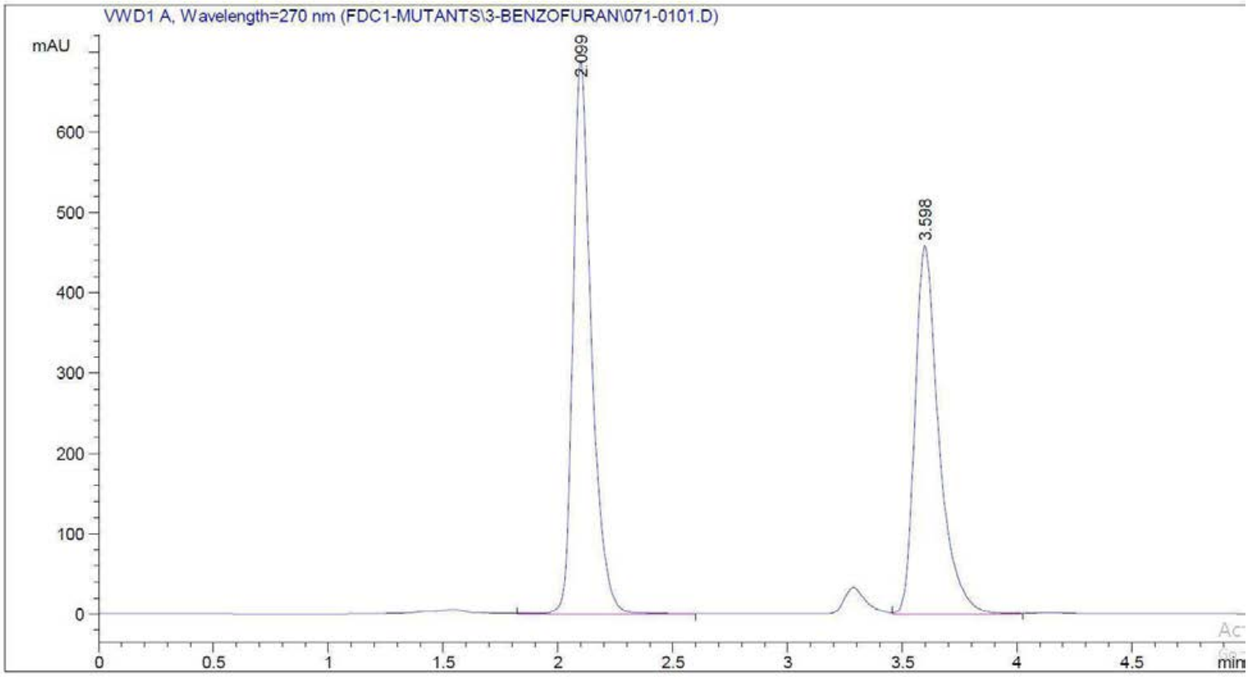

Figure S27. Chromatogram from the HPLC separation of benzalacetophenone and **1j**

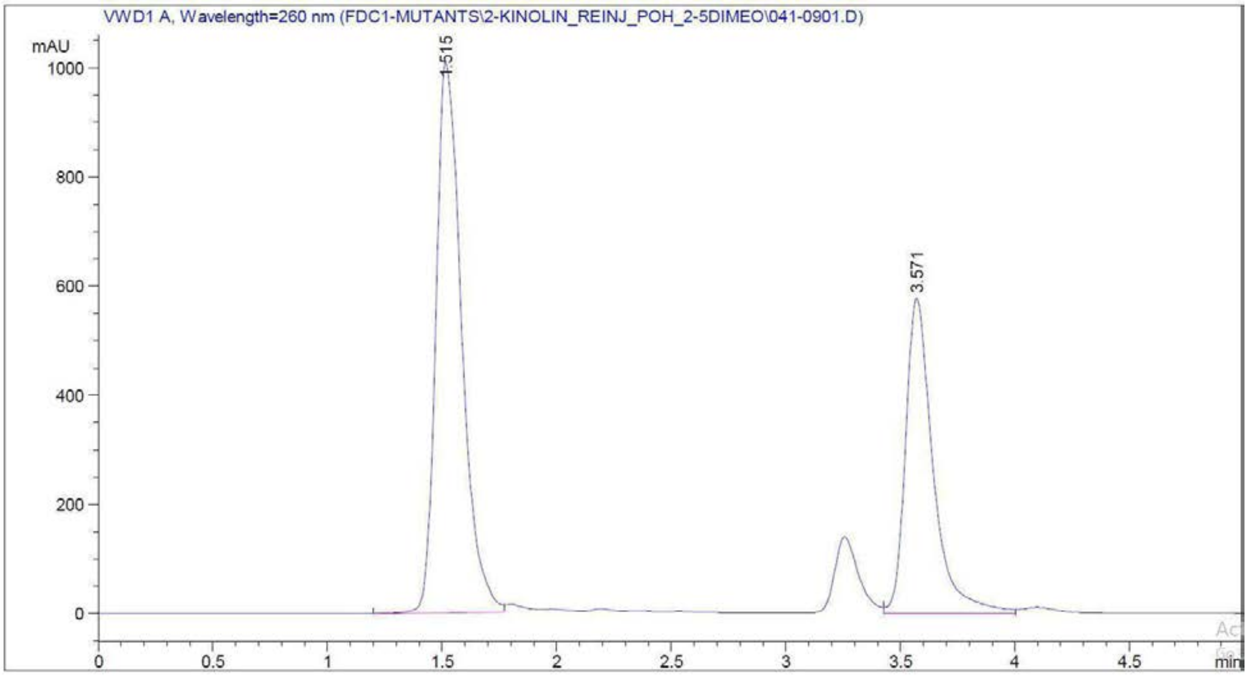

Figure S28. Chromatogram from the HPLC separation of benzalacetophenone and **1k**

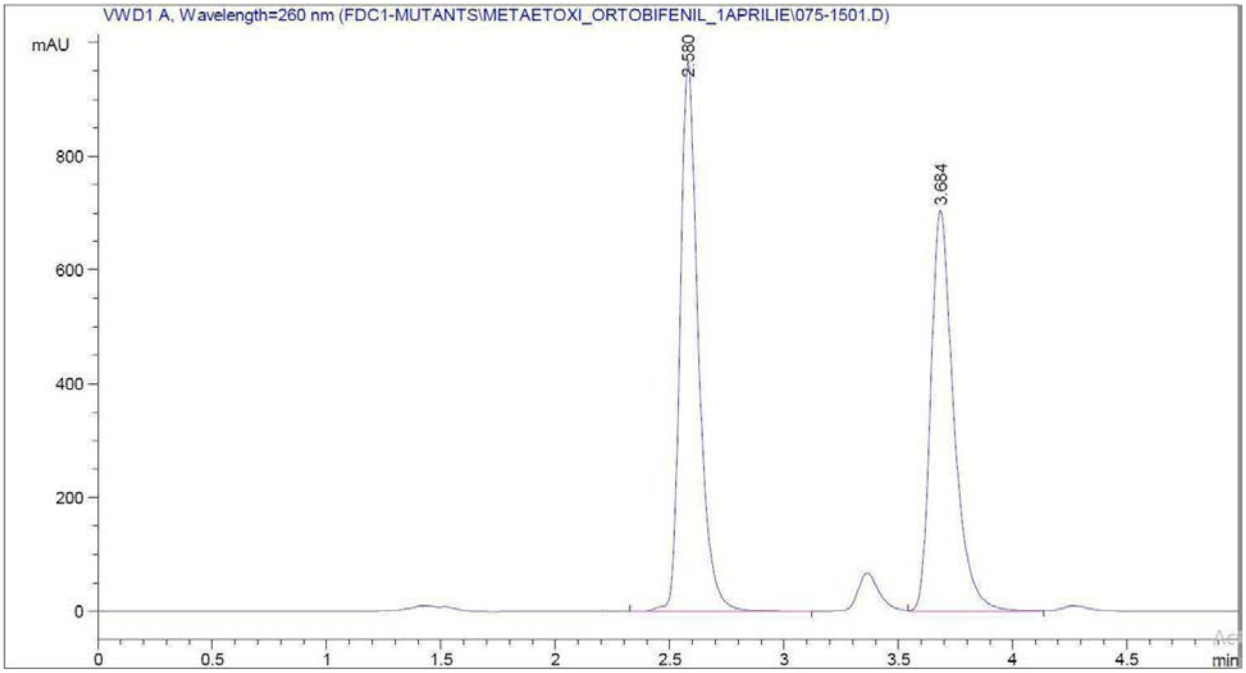

Figure S29. Chromatogram from the HPLC separation of benzalacetophenone and **1l**

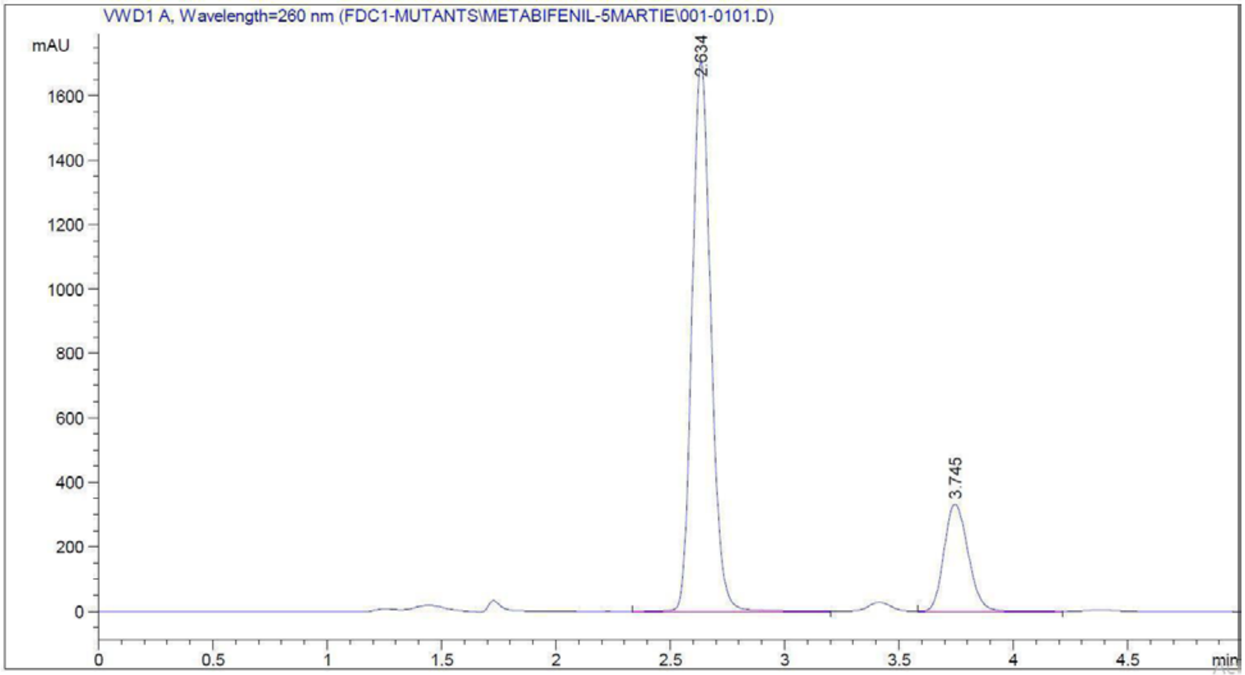

Figure S30. Chromatogram from the HPLC separation of benzalacetophenone and **1m**

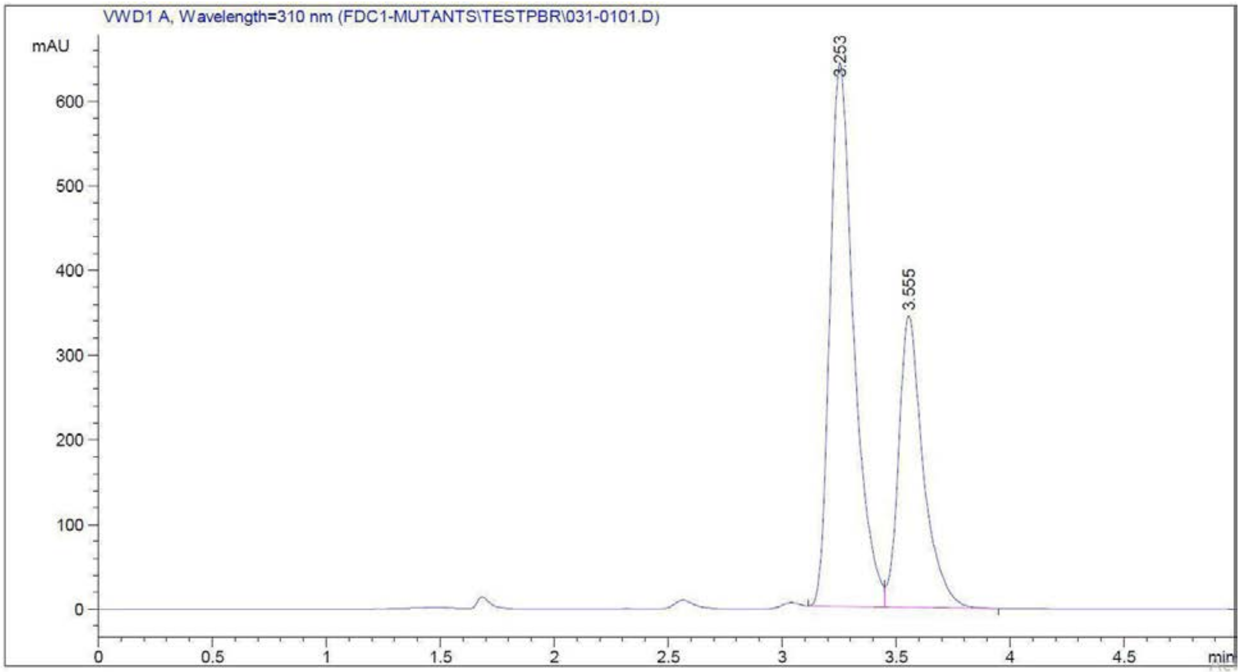

Figure S31. Chromatogram from the HPLC separation of benzalacetophenone and **1n**

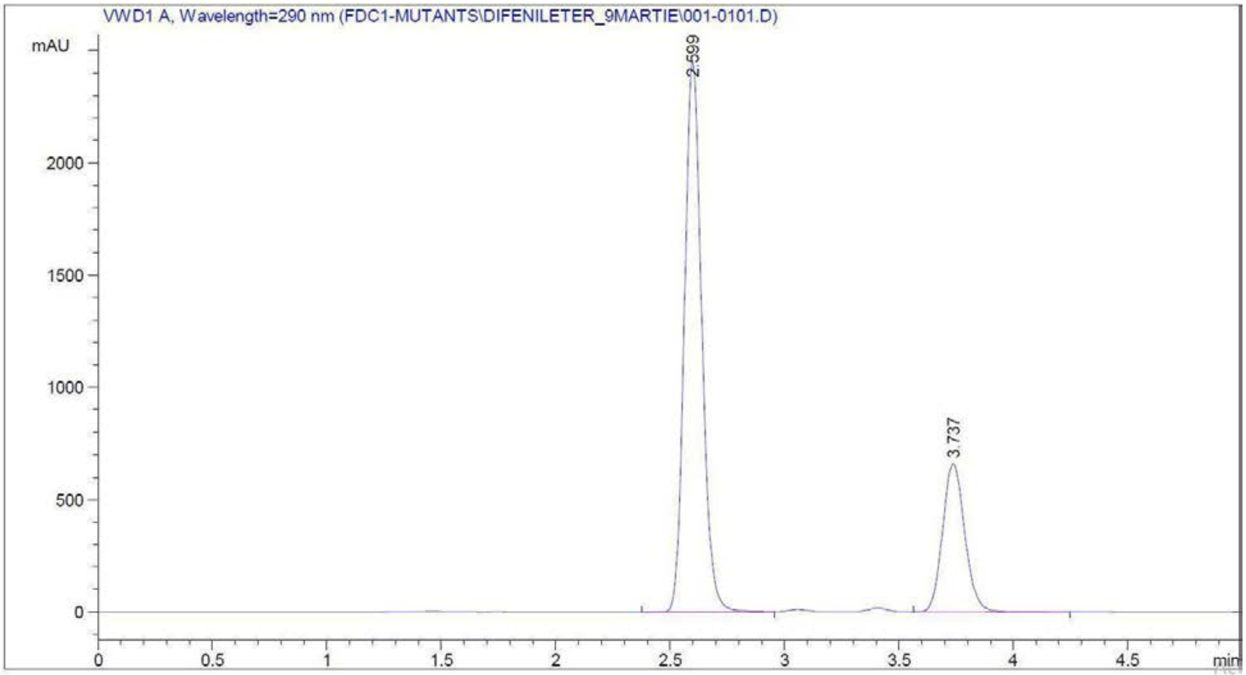

Figure S32. Chromatogram from the HPLC separation of benzalacetophenone and **1o**

8. Molecular modeling

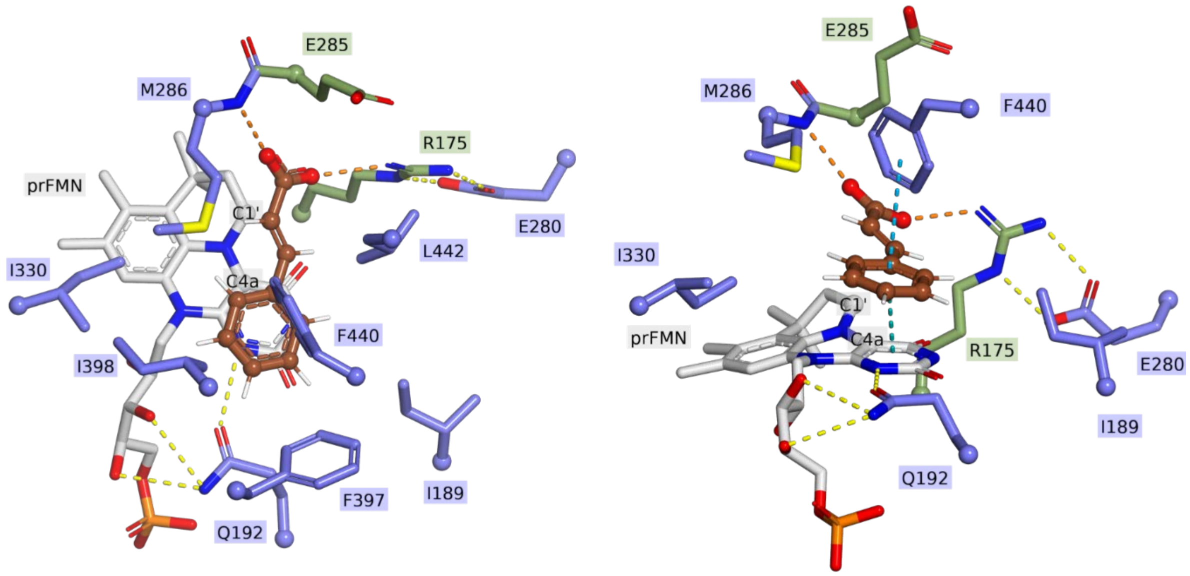

(a) (b)

Figure S33. Top view (a) and side view (b) of the active site with respect to the substrate plane: proper substrate binding requires the location of the α−β double bond of the substrate (highlighted in brown) in the proximity of carbon C1’ and C4a atoms of the prFMN cofactor. Hydrogen bonds between residues E280 and R175, as well as Q192 and the cofactor are shown as yellow dashed lines.

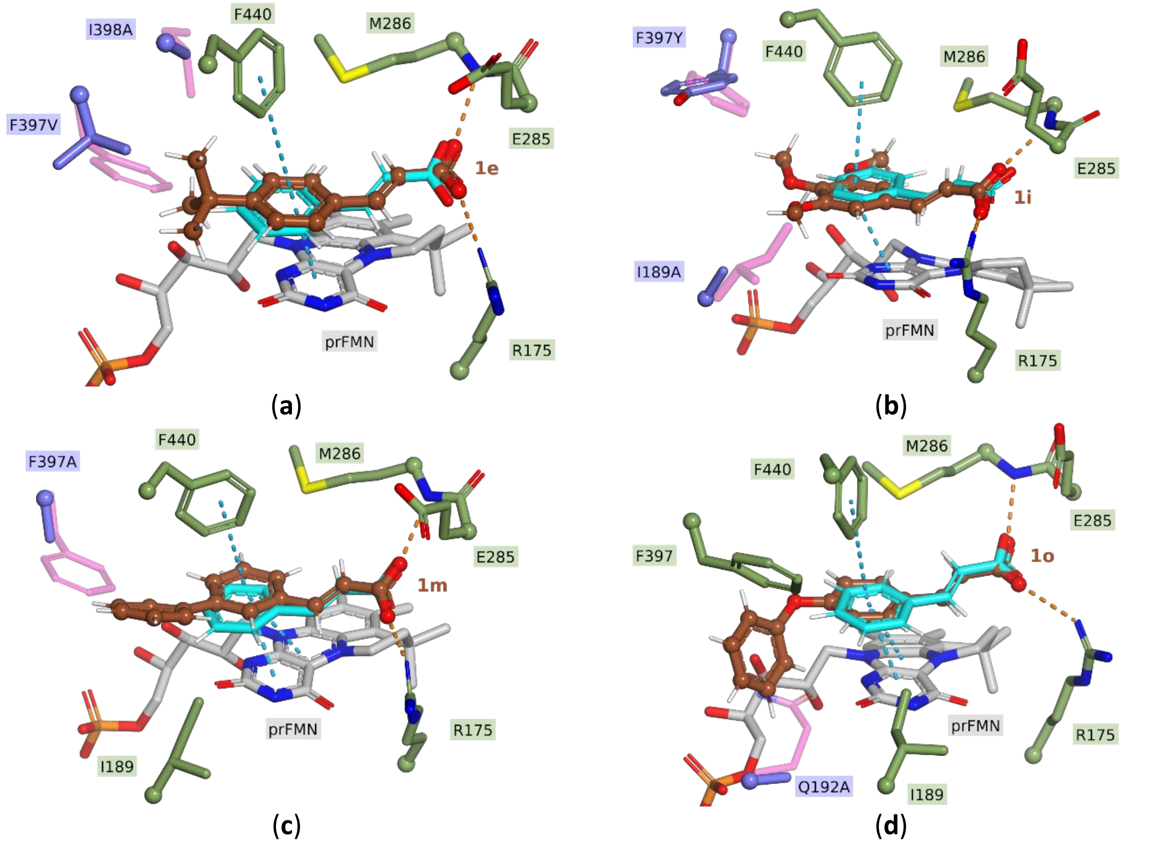

Figure S34. The optimized models presented in Figure 4 are overlayed with the optimized model of *trans*-cinnamic acid (highlighted in blue) docked in the *wt*-*Sc*FDC1.

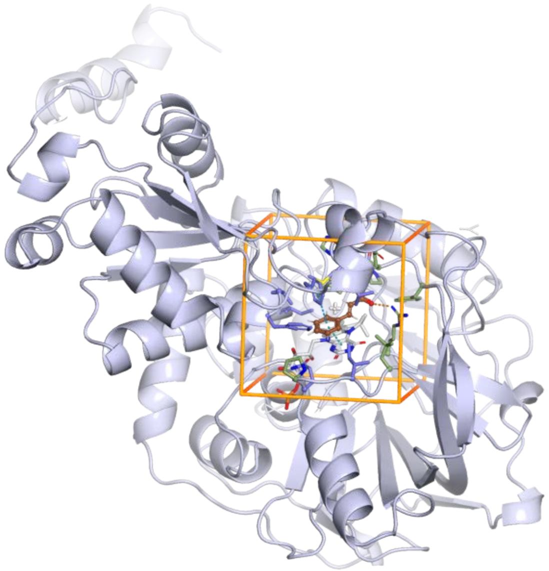

Figure S35. Cartoon representation of the *Sc*FDC1 crystal structure with PDB ID 4ZAC. The molecular docking calculations were performed in the search space represented by the orange cubic box.

9. References

[1] McKenna, R. & Nielsen, D. R. Styrene biosynthesis from glucose by engineered *E. coli*. *Metab. Eng.* **13**, 544-554, (2011), doi:10.1016/j.ymben.2011.06.005

[2] Lin, F., Ferguson, K. L., Boyer, D. R., Lin, X. N. & Marsh, E. N. G. Isofunctional enzymes PAD1 and UbiX catalyze formation of a novel cofactor required by ferulic acid decarboxylase and 4-hydroxy-3-polyprenylbenzoic acid decarboxylase. *ACS Chem. Biol*. 10, 1137–1144 (2015). doi.org/10.1128/AEM.03472-16

[3] Liu, H. & Naismith, J. H. An efficient one-step site-directed deletion, insertion, single and multiple-site plasmid mutagenesis protocol. *BMC Biotechnol*. 8, 91–101 (2008). doi.org/10.1186/1472-6750-8-91
